# Comprehensive analysis of microglia gene and subpathway signatures for glioma prognosis and drug screening: linking microglia to glioma

**DOI:** 10.1186/s12967-022-03475-8

**Published:** 2022-06-21

**Authors:** Chunlong Zhang, Jiaxin Zhao, Wanqi Mi, Yuxi Zhang, Xiaoling Zhong, Guiyuan Tan, Feng Li, Xia Li, Yanjun Xu, Yunpeng Zhang

**Affiliations:** 1 College of Bioinformatics Science and Technology, Harbin Medical University, Harbin, 150081 China; 2 Center of Cerebrovascular Disease, Zhuhai People’s Hospital, Zhuhai Hospital Affiliated With Jinan University, Zhuhai, 519000 China

**Keywords:** Microglia, ncRNA, Regulatory network, Brain disease, Biomarker

## Abstract

Glioma is the most common malignant tumors in the brain. Previous studies have revealed that, as the innate immune cells in nervous system, microglia cells were involved in glioma pathology. And, the resident microglia displayed its specific biological roles which distinguished with peripheral macrophages. In this study, an integrated analysis was performed based on public resource database to explore specific biological of microglia within glioma. Through comprehensive analysis, the biological characterization underlying two conditions, glioma microglia compared to glioma macrophage (MicT/MacT) as well as glioma microglia compared to normal microglia (MicT/MicN), were revealed. Notably, nine core MicT/MicN genes displayed closely associations with glioma recurrence and prognosis, such as P2RY2, which was analyzed in more than 2800 glioma samples from 25 studies. Furthermore, we applied a random walk based strategy to identify microglia specific subpathways and developed SubP28 signature for glioma prognostic analysis. Multiple validation data sets confirmed the predictive performance of SubP28 and involvement in molecular subtypes. The associations between SuP28 score and microglia M1/M2 polarization were also explored for both GBM and LGG types. Finally, a comprehensive drug-subpathway network was established for screening candidate medicable molecules (drugs) and identifying therapeutic subpathway targets. In conclusions, the comprehensive analysis of microglia related gene and functional signatures in glioma pathobiologic events by large-scale data sets displayed a framework to dissect inner connection between microglia and glioma, and identify robust signature for glioma clinical implications.

**Supplementary Information:**

The online version contains supplementary material available at 10.1186/s12967-022-03475-8.

## Introduction

Glioma is the most common primary brain tumors, which arises from glial cells within the central nervous system (CNS). The World Health Organization (WHO) classifies glioma on a grading scale of I, II, III, IV. Low Grade Glioma (LGG) typically ranges from grades I–III, while high grade glioma (HGG) are categorized as grades III–IV [1]. Glioblastoma multiforme (GBM) is a grade IV glioma subtype which highly invasive, making tumor recurrence certain even after a complete resection [2]. With the current standard of care, the median survival of patients diagnosed is approximately between 12 and 15 months. Thus, there is a substantial need to discovery of more effective therapies to improve patient outcomes.

Microglia, the resident macrophages of the CNS, are directly derived from yolk-sac erythro-myeloid precursor cells (EMP) during embryonic development [3, 4]. These mono-nuclear cells are 5–10% of cells and distributed throughout the brain, and their functions include regulating immune responses, supporting the homeostasis of the neurons, and maintaining the integrity of the blood–brain barrier (BBB) [4]. In the healthy brain, there is little turnover for microglia, however, the blood macrophages exhibit a high turnover rate. Although these two immune cell sub-populations were major included in the brain immune system, the different functions of microglia and peripheral macrophages were observed in brain pathology. And, the opposing effects of these two macrophage populations were reported in GBM tumors [5–7]. Furthermore, the genes/proteins used to distinguish these two populations are not exclusively expressed by either microglia or macrophages, but are only enriched, bringing more challenge for exploring the microglia specific biological roles.

Glioma microenvironment consists of various non-neoplastic cells that play an important role in tumor growth, progression, immune response evasion [8–10]. Among these cells, microglia is composed of about 30% of tumor mass [10] and displayed a close interaction with neoplastic cells. Also, the glioma cells secrete various cytokines acting as polarizing factors on the resident microglia. Recently, many single-molecular signatures which associated microglia and glioma cells were identified by low-throughput experiment strategy. In the study of Sarkar et al., the authors identified a novel factor Gas1 through which microglia arrest the growth of brain tumor initiating cells and displayed anti-tumor property [11]. And, study of Miyauchi et al. showed that Nrp1 could manipulate the immune functions of macrophages or microglia, and further exhibit its anti-glioma biological roles [12]. At the aspects of patient prognosis, it was indicated that M2-type microglia hold an unfavourable prognostic value in glioma by low-throughput methods [13]. However, most of these previous researches identified a limited number of microglia signatures at the gene level, and confirmed the glioma relevance using limited number of datasets. Also, the biological differences between microglia and macrophages, as well as cell-specific involvement in glioma events, were not performed.

Here, we performed a systematically integrated analysis based on several glioma-related microglia datasets. By comprehensively considering the difference between glioma-related microglia profiles and normal microglia profiles, as well as glioma-related microglia profiles and macrophage profiles, we explored the inner biological mechanisms involved in microglia at the glioma condition. In the meanwhile, the gene and subpathway-level signatures were specifically identified for microglia, and the closely connections between these signatures and glioma biological issues were revealed. Finally, a global drug-subpathway network was constructed for exploring the complex drug target relationship and identifying candidate treatment target regions. Based on comprehensive analysis of large-scale microglia and glioma data sets, several novel gene and functional signatures were identified to link microglia features and glioma biological events, with the potential of further clinical applications.

## Results

### Transcriptomic datasets integrated analysis reveals microglia specific biological roles

**Fig. 1:**
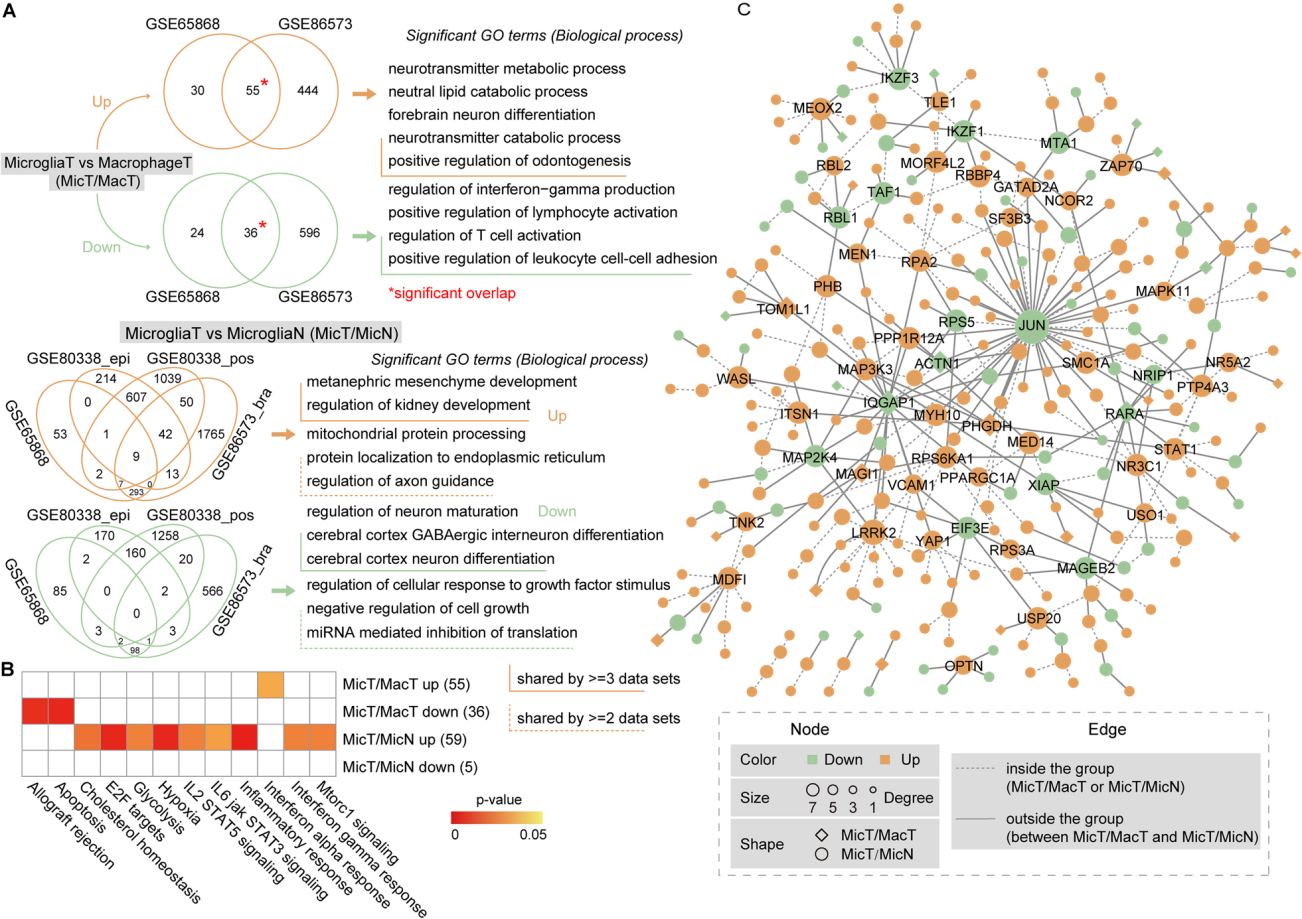
Microglia specific biological characterization and interaction network. **A** Two level of comparison analysis, microglia tumor vs macrophage tumor (MicT/MacT) as well as microglia tumor vs microglia normal (MicT/MicN), were performed. Bra indicated the brain tissue, epi indicated the epilepsy tissue, and pos indicated the postmortem tissue. The enrichment analysis for biological functions were performed based on signatures which existed at least two data sets (overlapping signatures). For MicT/MacT comparison, the functional analysis for the signatures which existed at least three data sets were further performed. **B** The associations between overlapping signatures (shared by three data sets) and known functional hall-markers derived from gene sets in The Molecular Signatures Database (MsigDB). The significant value was calculated by hypergeometric test. **C** The interaction network for gene signatures involved in MicT/MacT and MicT/MicN groups. The hub or key genes were shown in the network

To explore the specific biological roles of microglia at the glioma condition, we leveraged a series of published RNA sequencing and microarray datasets from brain microglia and macrophage populations isolated from glioma as well as normal samples (see “Materials and methods”). Considering microglia specific biological characterization, we performed two kinds of differential expression analysis, tumor microglia compared to tumor macrophage (MicT/MacT, two datasets) and tumor microglia compared to normal microglia (MicT/MicN, four datasets). We identified the differentially expression genes based on each datasets from MicT/MacT and MicT/MicN (Fig. 1A, see “Materials and methods”). Take the GSE86573 as an example (see Additional file 1: Fig. S1), many known signatures CXCL13, CCL1 and FFAR2 were differentially expressed in MicT/MacT. CXCL13 and CCL1 are all expressed in spinal astrocytes and previous studies have shown that CXCL13 played key roles in some microglia, macrophages, and endothelial cells after CNS infection [14, 15]. For the MicT/MicN results from GSE86573, HAS3, SOX10, AGT and SPAG6 were identified. SOX10, a transcription factor that interacts with Olig2, is important in non-neoplastic oligodendroglial development, and the dys-regulation of mRNA transcripts and protein expression are identified in a wider variety of CNS glial neoplasms [16, 17]. SPAG6 is incorporated into the central apparatus, showing its unambiguously important roles in stabilizing the axoneme [18]. There exists consistent differentially results among multiple datasets both for MicT/MacT and MicT/MicN (Fig. 1A and Additional file 2: Fig. S2). Notably, 9 up-regulated genes were shared by four datasets in MicT/MicN. As shown in Fig. 1A, the functional results in MicT/MacT contained neurotransmitter metabolic process, forebrain neuron differentiation, and positive regulation of odontogenesis (up-regulated) and immune related terms, such as regulation of T cell activation and positive regulation of lymphocyte activation (down-regulated). In MicT/MicN up-regulated results, some developmental and neural terms were involved, including metanephric mesenchyme development (shared by 3 datasets) and regulation of axon guidance (shared by 2 datasets). Neuron differentiation related terms were identified from MicT/MicN down-regulated results. Moreover, some tumor hallmarkers were also related with microglia specific genes, especially the MicT/MicN up-regulation genes (Fig. 1B).

To further explore the relationship among these microglia specific signature genes, we constructed a sub-network based on consistent differential expression genes which were shared by at least two datasets from MicT/MacT and MicT/MicN. Firstly, we obtained a high-quality interaction which were shared by at least two resources based on global protein–protein interaction (PPI) network derived from a previous study [19]. Then, a direct interaction among consistent signature genes based on PPI relation were constructed (Fig. 1C). The interactions with same directions within MicT/MacT or MicT/MicN were defined as “inside the group”, and the other interactions were defined as “outside the group”. As a result, many MicT/MicN signatures were located in the center of this sub-network and closely interacted with other genes, such as JUN, MAP2K4, and LRRK2. And some MicT/MacT signatures displayed “outside the group” interactions, such as PHGDH and MAGI1.

### 9 MicT/MicN core gene signatures in glioma biology

**Fig. 2:**
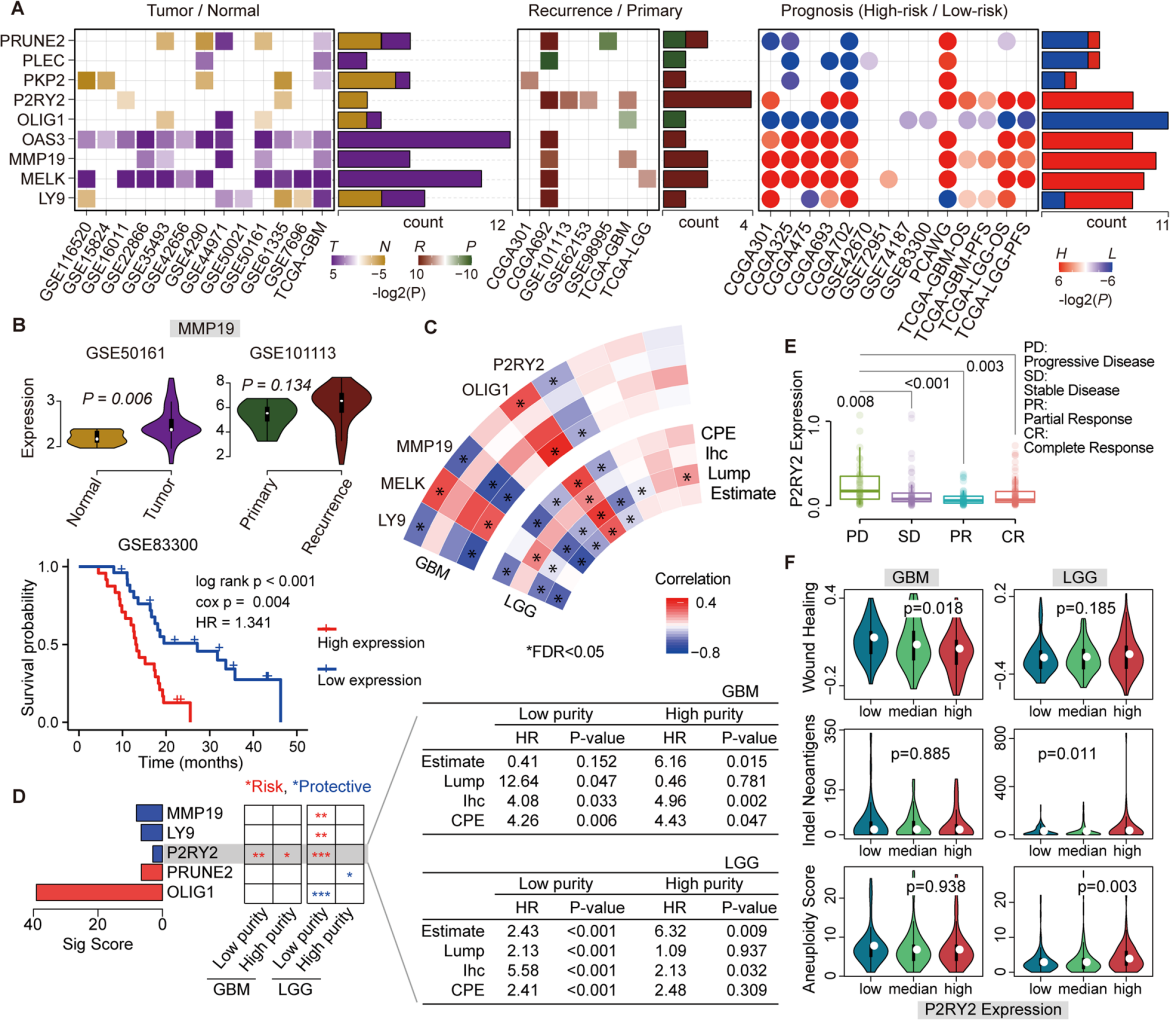
The 9 core MicT/MicN genes in glioma biology. **A** the performance of core genes in glioma Tumor/Normal, Recurrence/Primary, and High-risk/low-risk conditions. **B** An example for MMP19 gene from consistent signatures in GSE50161, GSE101113 and GSE83300. **C** the correlation map of core genes and tumor purity from four methods (Estimate, Lump, Ihc and CPE). The outside circle indicated GBM results and inside circle indicated LGG results. Red indicated positive correlation and blue indicated negative correlation. And star indicated significant results with FDR < 0.05. **D** The 5 genes with significantly positive and negative correlation were shown. And the prognostic performance of these genes in samples with high purity and low purity from CPE method were further evaluated. Red star indicated risk factors and blue star indicated protective factors. *0.05–0.01, **0.001–0.01, ***< 0.0001. The detailed survival results were displayed for P2RY2 gene. **E** The expression levels of P2RY2 signature in samples with different treatment results, PD: Progression Disease, SD: Stable Disease, PR: Partial Response, CR: Complete Response. **F** The associations between P2RY2 expression level and tumor characterization, including Wound healing, Indel neoantigens and Aneuploidy for both GBM and LGG types

The 9 common genes shared by four datasets in MicT/MicN were defined as MicT/MicN core genes, and we further dissected the involvement of these microglia specific genes in glioma biological issues, including tumor formation, recurrence and prognosis. A total of 34 public datasets derived from GEO, TCGA, CGGA and PCAWG were obtained (see “Materials and methods”, Additional file 15: Table S1). As shown in Fig. 2A, these 9 MicT/MicN core genes were closely associated with glioma biology. Some genes displayed consistent expression pattern across different glioma events. For example, OAS3 and MMP19 displayed higher expression levels in glioma samples compared to normal samples, recurrence samples compared to primary samples, high-risk group compared to low-risk group (Fig. 2B). MMP19, as a member of the MMPs family, had been reported to promote tumor growth, invasion, metastasis and chemoresistance [20, 21]. And previous study had revealed that glioma patients with higher expression of MMP19 protein had shorter overall survival times [22], which was consistent with our findings. At the aspect of recurrence, the P2RY2 gene displayed higher expression level within three datasets, which was also consistent with the prognostic results.

To test the cell source from immune cells or tumor cells, we further explored the associations between these MicT/MicN core genes and tumor purity, where were estimated by four methods based on TCGA GBM and LGG samples [23]. Among these genes, five genes displayed significant associations with tumor purity (see Fig. 2C and D). Notably, MMP19, LY9 and P2RY2 displayed negative association GBM and LGG purity. Previous researches had found that tumor purity was a prognostic factor for glioma samples [24]. And then, we divided all samples into low-purity and high-purity samples, and the prognostic performance of purity associated genes within these two groups were tested. As shown in Fig. 2D, most genes displayed good predictive performance in LGG than GBM, reflecting the high-grade of GBM. Still, for both GBM and LGG samples, P2RY2 displayed consistent risk predictive performance for both high-purity and low-purity groups, based on four methods (Fig. 2D). Furthermore, we tested the involvement of these P2RY2 signature in clinical response within LGG type with adequate sample number (Fig. 2E). Consistent with the prognostic performance, the risking genes P2RY2 also displayed higher expression level in PD group than SD group (P-value = 0.008), PR group (P-value < 0.001) and CR group (P-value = 0.003). To explore the biological roles, it was observed that the expression level of P2RY2 was associated with wound healing function in GBM type and indel neoantigens and aneuploidy functions in LGG type (Fig. 2F).

### Identifying microglia specific subpathways and crosstalk network

Subpathways, regions within the whole pathways, displayed closely association with disease formation and progression, which were revealed by our previous studies [25, 26]. We further developed a novel framework to identify microglia specific subpathways based on both MicT/MacT and MicT/MicN conditions (see Additional file 3: Fig. S3, Materials and Methods). In briefs, we firstly applied network-based random walk algorithm to optimize candidate genes for consistent up-regulated and down-regulated signatures of MicT/MacT and MicT/MicN groups. To validate the robust of random walk results, we obtained another independent data set (GSE29949) to test the results of top 10 genes from MicT/MacT group. As shown in Additional file 4: Fig. S4, nine of ten up-regulated genes displayed significant higher expression level in microglia than other cell types, including monocyte and dendritic cell. And for down-regulated genes ranked by random walk, consistent expression patterns were also observed. Secondly, we obtained subpathway list and calculated the subpathway score by comprehensive considering different dysregulated impacts. And a random strategy (5000 times randomization) was applied to evaluate the significance of all subpathways. As a result, a total of 1/34 up-regulated/down-regulated subpathways from MicT/MacT, and 18/46 up-regulated/down-regulated subpathways from MicT/MicN were identified (see Additional file 5: Fig. S5). Notably, four subpathways were shared by MicT/MacT down-regulated and MicT/MicN down-regulated results, including four subpathways from Regulation of Action Cytoskeleton (Path: 04810) pathway. Finally, based on four types of microglia specific subpathways, we utilized gene overlapping to construct a subpathway crosstalk network. As shown in Additional file 6: Fig. S6, the subpathways with same dysregulated direction displayed closely interactions and most of these subpathways (68/82) were down-regulated. Many subpathways from Axon guidance, Natural killer cell mediated cytotoxicity, and Leukocyte transendothelial migration were the hub subpathways, and four shared subpathways were located in the center of this network.

### Microglia subpathways associated with glioma biology

**Fig. 3:**
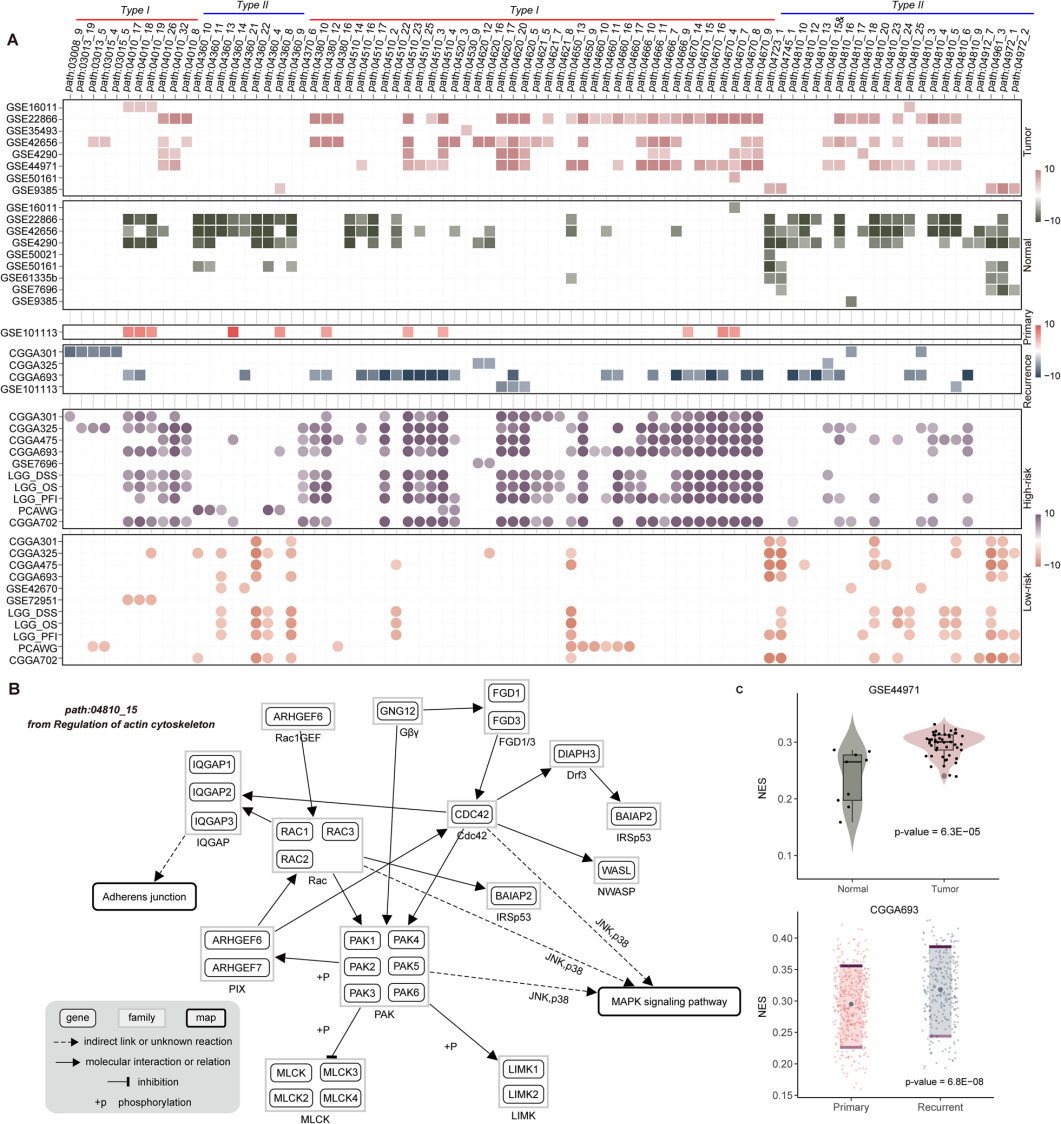
The associations between microglia subpathways and glioma biology. **A** The associations of genes involved in subpathways and glioma Tumor/Normal, Recurrence/Primary, and High-risk/low-risk conditions. The data analyzed were the same as Fig. 2A. And two types of subpathways were defined based on the association with glioma biology. **B** A subpathway graph of path: 04810_15 in KEGG database. **C** The subpathway activity calculated by ssGSEA method in two GEO datasets, GSE44971 (Tumor/Normal) and CGGA693 (Primary/Recurrence)

Based on the available glioma data sets used in Fig. 2A, we further explored the associations between microglia specific subpathways and glioma biological events (see “Materials and methods”). As shown in Fig. 3A, all these subpathways displayed two types of expression patterns. Most subpathways from Type I displayed risk expression pattern in glioma formation, recurrence and prognosis, whereas the subpathways from Type II displayed protective pattern. Type I subpathways were majorly derived from Immune system and Cellular community classes, and Type II subpathways were derived from Cell motility, and Development and regeneration classes (see Additional file 7: Fig. S7). At the glioma biology, these subpathways displayed closely associations with tumor formation, especially the prognosis condition. Take the path: 04810_15 (from Type II) as an example, this subpathway displayed higher expression pattern in tumor samples not normal samples, whereas the other subpathways from the same whole pathway (path: 04810) displayed consistent higher expression pattern in normal samples. Next, we calculated the NES score for this subpathway by using the ssGSEA method (see Fig. 3B and C). Similar as the expression pattern from previous result, this subpathway indeed displayed higher functional activity in tumor samples (GSE44971, P-value = 6.3E-05), and higher functional activity in recurrence samples (CGGA693, P-value = 6.8E-08).

### A subpathway-based risk signature for glioma prognosis

**Fig. 4:**
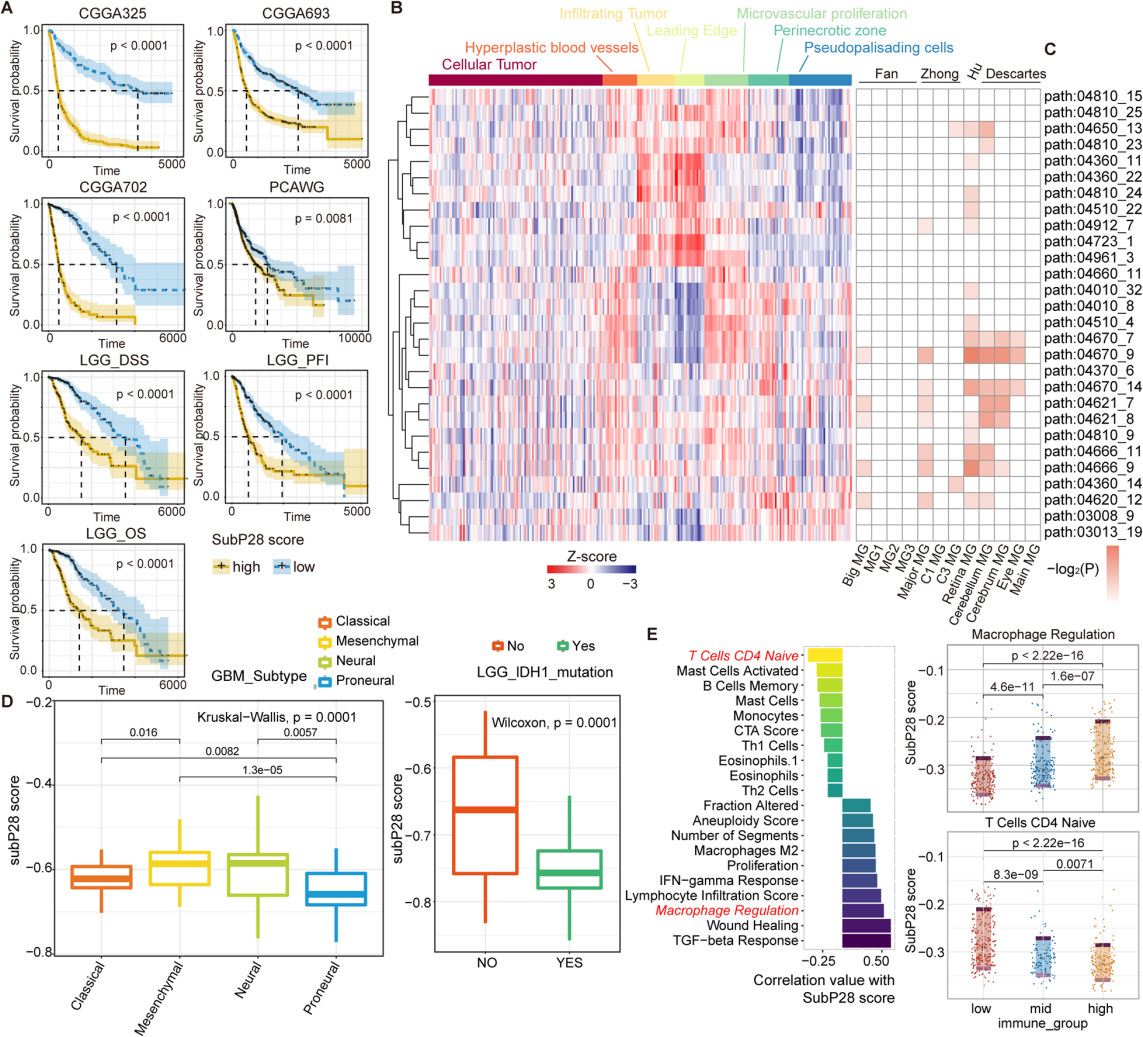
The predictive performance of SubP28 signature and immune infiltration association. **A** The K-M plot of SubP28 signature in independent testing sets from CGGA, PCAWG, and TCGA databases. **B** The SubP28 score in the Ivy Glioblastoma Atlas Project. Each column annotates subpathway activity in RNA-seq of an anatomically defined tumor compartment. **C** The associations between SubP28 signature and other microglia signatures of C8 gene sets from MsigDB database. **D** The associations of SubP28 score with GBM samples with different molecular sub-types and LGG samples with IDH1 mutation event. **E** The associations between SubP28 score and immune infiltration characterization

Our findings revealed that subpathways in the network were closely related with glioma prognosis, especially LGG type (see Fig. 3A). Therefore, we further performed a lasso-based strategy to construct a prognostic model based on 1185 glioma samples of integrated microarray datasets (see “Materials and methods”). As a result, 28 microglia specific subpathways with best predictive performance were identified and defined as SubP28 signature (see “Materials and methods”, Additional file 8: Fig. S8). And the detailed coefficient information of SubP28 signature was provided in Additional file 16: Table S2. For the seven independent testing sets from CGGA, TCGA and PCAWG database, the samples with higher SubP28 score displayed consistent poor survival results than samples with lower score (see Fig. 4A). When considering the aspect of tumor purity, we observed that the SubP28 score of LGG samples was negatively related with tumor purity, however the GBM samples did not (see Additional file 9: Fig. S9A and B). Within the low-purity and high-purity LGG samples, the SubP28 score also predicts the patient survival with significant results (Additional file 9: Fig. S9C and S9D). In the meanwhile, the SubP28 displayed independently predictive performance when considering other clinical factors such as grade, age and IDH1 mutation (see Additional file 9: Fig. S9E).

Furthermore, we quantified our SubP28 signature in data from the Ivy Glioblastoma Atlas Project (IGAP), which performed RNA-seq on microdissections of glioma anatomical structures from hematoxylin and eosin (H&E) staining [Ivy Glioblastoma Atlas Project. http://glioblastoma.alleninstitute.org]. The higher functional activity of SubP28 signatures were enriched in samples from the leading edge of invading glioma and in infiltrating tumor regions. And these subpathway signatures displayed lower activity in cellular tumor and pseudopalisading cells (see Fig. 4B). To test the microglia association of our SubP28 signature, we further obtained several microglia related sets from MsigDB database and calculated the significance of overlapping using the hypergeometric test. As shown in Fig. 4C, the SubP28 signature displayed close association with other microglia (MG) signature.

The glioma samples with different subtypes displayed different prognostic results, and the difference of the SubP28 score between samples with GBM molecular subtypes was also observed (see Fig. 4D). The samples with mesenchymal and neural types exhibited higher SubP28 score than other types. For LGG, we further explored the associations between SubP28 score and IDH1 mutation. The results showed that LGG samples with IDH1 wide type displayed higher SubP28 score. In the meanwhile, the SubP28 displayed significant predictive performance in LGG samples with IDH1 wide type (see Additional file 10: Fig. S10). The correlation analyses revealed that the SubP28 score was positively related with macrophage regulation function, and negatively related with CD4 T cells (see Fig. 4E). Notably, the SubP28 score was also positively related with Macrophage M2 type (a tumor promoting factor), which was consistent with its risk maker in the prognostic results.

### SubP28 related with microglia states for both GBM and LGG

**Fig. 5:**
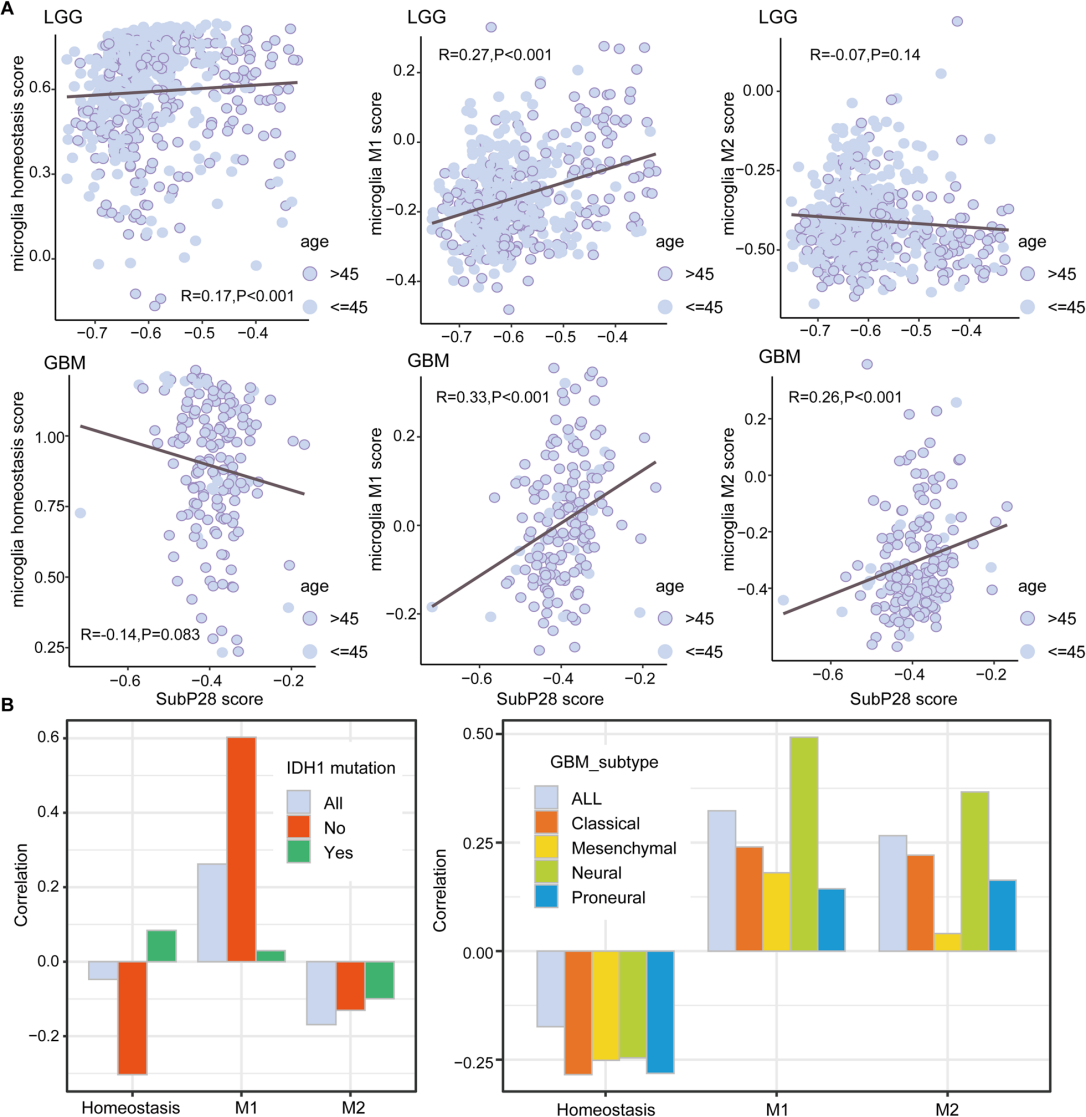
**A** The association of SubP28 score and microglia homeostasis, M1 and M2 scores for both GBM and LGG types. **B** The correlation of SubP28 score with microglia conditions when considering GBM molecular subtypes, and LGG IDH1 mutation conditions

To further explore the associations between SubP28 signature and microglia cell state, we calculated the microglia score for TCGA GBM and LGG samples by using the ssGSEA method. And the homeostasis marker (CX3CR1, CSF1R, P2RY12, and TMEM119), M1 marker (IL12B, IL12A, IL23A, TNF, NOS2, and CXCL10) and M2 marker (RETNLB, IL10, ARG1, and MRC1) were utilized for correlation analysis. As shown in Fig. 5A, the SubP28 score of LGG samples were positively related with microglia homeostasis condition, which was not for GBM samples. And for microglia M1 and M2 conditions of GBM samples, the SubP28 score was positively related with microglia activity. It implied the possible mechanism that GBM was majorly consisted of M1/M2 microglia than ones of LGG samples, which was consistent with previous findings. When considering the molecular subtypes and IDH1 mutation, we further observed that M1 and M2 specific associations (see Fig. 5B). It was shown that the SubP28 score displayed positive correlation with M1 markers within LGG samples without IDH1 mutation. For GBM molecular subtypes, the positive correlations with M1 and M2 markers were speciality involved in neural types. Furthermore, we utilized two single-cell RNAseq data sets of glioma to test the SubP28 signature in multiple cells. And it was observed that the SubP28 signature was specific involved in microglia and oligodendrocytes (GSE84465 and GSE89567, see Additional file 11: Fig. S11).

### Drug-subpathway network reveals novel treatment strategies

To predict the drug sensitivity in high SubP28 or low SubP28, we systematically evaluated the association between SubP28 score and response sensitivity to anti-neoplastic agents. We obtained the drug response data from three resources, (1) Genomics of Drug Sensitivity in Cancer (GDSC) database for GBM and LGG cell lines [27], (2) Human Glioma Cell Culture (HGCC) cohort, which reported an integrated pharmacogenomic analysis of 100 patient-derived GBM cell cultures treated by 1544 drugs [28], (3) the predicted results from Elastic Net prediction model (LENP), which predict the response sensitivity to antineoplastic compounds for TCGA tumor types, including GBM and LGG [29]. Using the half maximal inhibitory concentration (IC50) value, we calculated the correlation relationship between IC50 of drug/molecules and SubP28 score. Combining the correlation results and drug treatment information, we obtained two candidate drug sets, (1) the drugs displayed higher response sensitivity in cell lines with high SubP28 score, (2) the drugs displayed higher sensitivity with low SubP28 score (see Additional file 12: Fig. S12). Among the drug set i, Lapatinib, a drug for BRCA treatment was identified as sensitive molecule within high SubP28 score group. In the meanwhile, Gemcitabine, Methotrexate, and 5-Fluorouracil were identified to be more sensitive in cases with low SubP28 score.

**Fig. 6:**
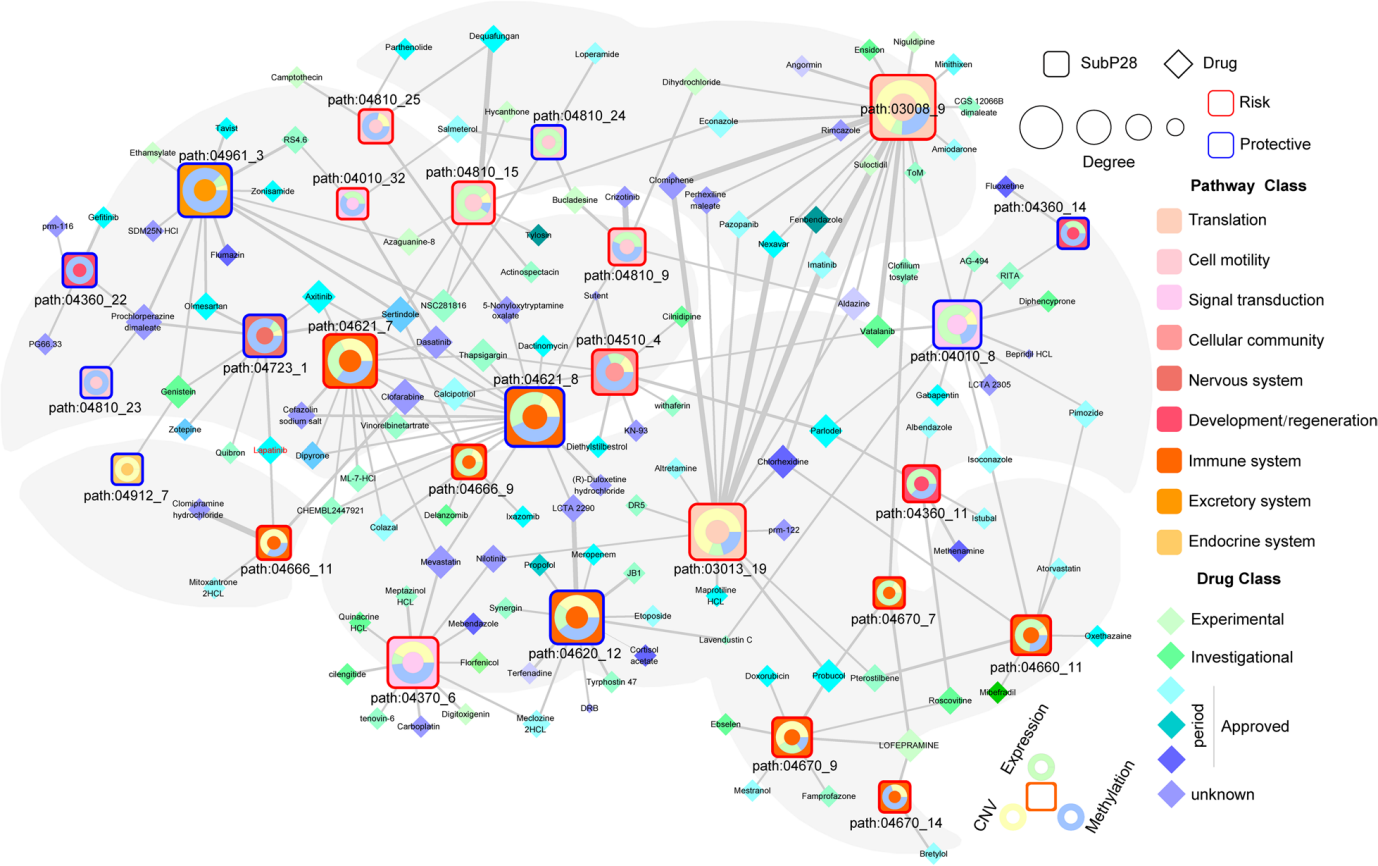
The drug-subpathway network in HGCC. The network shows the subpathways (squares) that could be targeted by drugs (diamonds) based on three level of omics data from HGCC database. The associations between genes within subpathway and three level datas are denoted by colors in the ring chart. The color of subpathway and drug indicates the pathway class and drug class. The subpathway classification (risk or protective) is denoted by the color of the square border. The edge width indicated the number of associations between drug and corresponding subpathway at three levels. The size of subpathways increase with the degree which reflects the associations with drugs

To explore the detailed associations between antineoplastic compounds and 28 microglia subpathways, we constructed a multi-omic integrated network based on HGCC resource (see “Materials and methods”). As shown in Fig. 6, many microglia specific subpathways were targeted by many candidate drugs from three omics levels, such as path: 03013_19, path: 03008_9, path: 04621_7, and path: 04010_8. And most of these subpathways belonged to the risk subpathway set within the SubP28 model. And the different subpathways (_7 and _8) from the same pathway (path: 04621) displaying opposite prognostic pattern also shared some drugs, such as Clofarabine, Dasatinib, and Thapsigargin. Notably, the path: 04010_8 was enriched by GBM related genes, and GBM mutation genes, including RAC2, NFKB1, RAC1 (disease gene) and MAP4K3, and MAP3K1 (mutation). Also the risk path: 04670_9 was also enriched by GBM related genes, showing its potential roles as the treatment target in the clinical trails.

## Discussion

In this study, we performed comprehensive analysis for identifying microglia specific gene and subpathway signatures and exploring their associations with glioma biology based on large-scale transcriptomic datasets. Among the gene signatures, P2RY2 displayed predictive performance in glioma patients when considering other clinical factors such as tumor purity. Furthermore, the subpathway-level risk model, subP28, were constructed as a functional signature for predicting patient prognosis and clinical treatment response. Finally, the complex associations between these subpathways and candidate drugs were explored. All these findings indicated inner evidence for connecting microglia and glioma, also provided core signatures for glioma prognostic, drug response and clinical treatment guidance.

Among the MicT/MicN group, nine consistent up-regulated genes were identified by four datasets. And these genes displayed closely associations with glioma formation, recurrence, and prognosis events. Notably, there exists connection between P2RY2 signature and glioma, both in low-purity and high-purity samples. Several subtypes of the P2Y receptors and their functions have been identified in microglia. A previous study found that upregulation of the P2RY2 is detected in macrophage/microglia after spinal cord injury [30]. P2RY2 expression was also found to be increased in activated microglia. In mice model, P2RY2 is an important receptor for the recruitment and activation of microglia [31]. Among the purinergic receptors that are activated by ATP, P2RY2 could regulates cell proliferation in various tumors, such as lung and bladder cancer [32, 33]. Moreover, P2RY2 up-regulation occurs in response to stress or injury in blood vessels and epithelium, and has been linked to the stimulation of smooth muscle growth [34, 35]. Thus, the novel mechanisms of P2RY2 up-regulation and function in the nervous system warrant further investigation, providing new strategies for the treatment and management of corresponding brain diseases.

Based on the global random walk, the microglia specific subpathways were identified. The subpathways, different regions within whole pathway, displayed more specificity than whole pathway and more robust than gene-level signature. As shown in Fig. 3A, the different subpathway signatures derived from the same pathway indeed displayed different patterns, such as Path: 04810_15. Similarity, we also observed that this subpathway displayed higher activity in samples with mesenchymal type (Additional file 13: Fig. S13A) and samples with IDH1 wide type (Additional file 13: Fig. S13B). And the similar results were also shown in another independent datasets (Additional file 13: Fig. S13C).

Glioma cells secrete various cytokines and chemokines acting as chemo-attractants and polarizing factors on the resident microglia [10]. In tumor microenvironment, infiltrating microglia adopt different activation states between antitumor M1 and protumor M2 phenotypes, and these functional phenotypes are defined by differential expression of surface markers, secreted cytokines, and roles in immunoregulation [36]. Activated microglia assume the M1 phenotype characterized by the expression of STAT1 and are capable of stimulating antitumor immune responses by presenting antigens to adaptive immune cells, producing proinflammatory cytokines, and phagocytosing tumor cells. In comparison, the alternatively activated pathway, M2 is characterized by expression of the scavenger receptors, intracellular STAT3 and the production of immunosuppressive cytokines. M2 polarization prevents the production of cytokines required to support tumor-specific CD8^+^ T, CD4^+^ Th1, and Th17 cells and promotes the function of tumor-supportive CD4^+^ regulatory T cells [37]. Recent studies indicate that glioma cells induce a mixed population of GAMs expressing both M1-and M2-related molecules [38]. And in this study, the microglia specific SubP28 score was positively related with macrophage M2 condition, which was also the high risk factor in glioma prognosis (see Fig. 4E). The concept of GAMs playing a key role in glioma pathobiology has been verified in recent studies that demonstrated reduction of glioma growth after macrophage ablation or pharmacological inhibition [39]. Furthermore, we systematically compared our SubP28 signatures with several glioma functional sets [40–42]. As shown in Additional file 14: Fig. S14, it was shown that SubP28 shared many overlapping genes with microglia/macrophage gene sets, and glioma NPC2 & MES conditions. In the meanwhile, these exists no associations with SubP28 signatures and cell cycle characterization (G1/S and G2/M sets).

To comprehensively explore specific biological roles involved in microglia not in macrophage, we searched and obtained datasets from brain tissues which contained both microglia/macrophage and glioma/normal information. Some datasets derived from blood or other fluid tissue were removed, which provided limited resource for resident microglia functional exploration. With the development of more available datasets, the gene and subpathway signature will be further confirmed as their clinical roles. We conclude that engaging microglia signatures identified, reflecting the immune roles within the microenvironment of glioma, will lead to novel therapies that improve the outcome of patients suffering from this terrible disease.

## Materials and methods

### Gene expression data sets

In this study, available gene expression datasets were identified from Gene Expression Omnibus (GEO) database by specifically choosing only studies that performed gene expression analysis of both resident microglia and macrophages from brain tissue in glioma condition. Datasets utilized for microglia integrated analysis included GSE65868, GSE86573 and GSE80338. The disease condition (glioma or normal) and cell populations (microglia or macrophage) were obtained from the previous researches. Two species involving mouse and human were included, and gene IDs conversation were performed using R package *org.Hs.eg.db*. Within the GSE86573, the blood tissue was not considered, and the samples from brain tissue were regarded as normal samples. Within the GSE80338, epilepsy and postmortem samples were respectively regarded as normal samples.

To explore the biological roles of microglia gene or functional signatures in glioma pathology, large amount of gene expression data sets with glioma clinical information were obtained from GEO, The Cancer Genome Atlas (TCGA), Chinese Glioma Genome Atlas (CGGA), and Pan-Cancer Analysis of Whole Genome (PCAWG) databases. And a total of 26 data sets were included. These data sets contained three kinds of glioma events, including glioma and normal samples, recurrence and primary samples, and samples with prognostic information. The detailed description of all gene expression data sets mentioned above were given in Additional file 15: Table S1.

### Differentially expression analyses

We utilized two methods to respectively performed differentially expression analyses for RNA sequencing and microarray datasets. For RNA sequencing datasets (GSE86573 and GSE80338), we utilized R *DEseq2* package to identify differentially expression genes based on the raw count matrix. For one microarray dataset (GSE65868), we identified the differentially expression genes based on FPKM expression matrix by integrating fold change and T-test methods. For all datasets, the differentially expression genes were obtained by absolute log2-based fold change > 1.5 and false discovery rate (FDR) or adjusted P-values < 0.05.

### Functional exploration for microglia specific genes

Based on the consistent differential expression genes shared by 2 MicroT/MacroT datasets, and 2 or 3 MicroT/MicroN datasets, we further performed functional enrichment analysis by using the R *clusterProfiler* package [43]. And the Gene Ontology (GO)—Biological Process (BP) terms were considered. Also, the tumor hallmark gene sets were obtained from the molecular signature database (MsigDB) for functional association analysis. And hypergeometric distribution test was used to evaluate the associations between consistent genes and known hallmarkers, and the P-values were calculated as follows:

$$P = 1 - \sum\limits_{x = 0}^{r - 1} {\frac{{\left( {\begin{array}{*{20}c} t \\ x \\ \end{array} } \right)\left( {\begin{array}{*{20}c} {m - t} \\ {n - x} \\ \end{array} } \right)}}{{\left( {\begin{array}{*{20}c} m \\ n \\ \end{array} } \right)}}}$$

where m was the number of the human whole genome, and t was the number of genes included in one hallmark gene set. The number of consistent signature genes was n, and *r* genes out of n genes were included in the hallmark gene set.

### A novel framework for identifying microglia specific subpathways

For exploring the biological roles of microglia at the functional level, we developed a novel framework to identify microglia specific subpathways. As shown in Additional file 3: Fig. S3, we firstly obtained a high-quality PPI network from a previous study [19], which displayed protein–protein interaction from at least two data resources. Based on the global network, we further performed a global impact analysis to rank candidate mRNAs by using random walk algorithm [44]. And the consistent gene signatures of microglia, shared by two data sets in MicT/MacT group and three data sets in MicT/MicN group, were regarded as seed nodes. As a results, a total of 55/36 up-regulated/down-regulated genes from MicT/MacT group, and 59/5 up-regulated/down-regulated genes from MicT/MicN group, were respectively annotated into network as seed. And, the random walk algorithm was performed four times to evaluate the global impact of seed nodes at different aspects as follows:

$$P^{t + 1} = \left( {1 - r} \right)WP^{t} + rP^{0}$$

where *W* was the column-normalized adjacency matrix of the global network, which consisted of 0 and 1. P^*t*^ was a vector, in which a node in the network held the probability of finding itself in this process up to step *t*. The initial probability vector, *P*^0^, was constructed in such a way, where equal probabilities were assigned to all seed nodes and the sum of their probabilities was equal to 1. Additionally, the restart of the walker at each step was the probability, *r* (*r* = = 0.7). When the difference between P^*t*^ and P^t+1^ fell below 10^−6^, the probabilities reached a steady state. Finally, each gene in the network was given a score according to the values in the steady-state probability vector, *P*^∞^. After random walk algorithm, each candidate gene get four scores (score_up and score_down) from both MicT/MacT and MicT/MicN groups.

Then, we obtained subpathway list from R *subpathwayMiner* package [26], which contained at least three gene components from 1773 subpathways. For each subpathway, we calculate the subpathway score based on the gene score from random walk analysis. And, the formula was provided as follow:

$${\text{Score}}_{{{\text{subpath}}}} = \overline{{Score_{up} }} - \overline{{Score_{down} }}$$

where $\overline{{Score_{up} }} /\overline{{Score_{down} }}$ was respectively the mean score of genes within one subpathway for MicT/MacT and MicT/MicN groups. For each subpathway, we further performed 5000 random perturbation by random assigning score which was equal to original gene number to calculate the significance as follows:

$${\text{P - }}value_{{{\text{subpath}}}} = \frac{{\left| {Score_{Random} > Score_{True} } \right|}}{5000}$$

where was the true subpathway score and was the random results. From this analysis, the subpathways with P-value < 0.001 were identified as the microglia specific subpathways. Based on these subpathways, a subpathway network was also constructed if any two subpathways shared more than seven gene components.

### The associations between microglia subpathways and glioma biology

For all glioma data sets, we took three types of comparison into consideration, including tumor compared to normal samples, recurrence compared to primary samples, high-risk compared to low-risk samples. And the R *limma* package was utilized to perform the differentially expressed analysis. For this analysis, we identified differentially expressed genes (up-regulated and down-regulated both considered) based on the adjusted P-value < 0.05 and absolute log2-based fold-change > 0.5. And the univariate cox analysis was performed to identify high-risk or low-risk genes based on the P-value < 0.01 and absolute HR > 1. And the data sets without significant gene results were removed. And then, the overlapping between genes within subpathway and up-regulated (high-risk) or down-regulated (low-risk) genes from each data set were evaluated using the hypergeometric test method. And P-value < 0.05 was considered as the significant associated result.

### Identification of subpathway prognostic model

We constructed a prognostic model by utilizing expression profiles of microarray platform as the training set. There were systematic deviations between the microarray datasets generated by different laboratories at different times. Therefore, we firstly utilized Combat function in the R *SVA* package to eliminate the batch effect and formed a merged training set. Based on the training set, we further calculated the NES enrichment score for each subpathway. And then, a generalized linear model by a maximum likelihood estimation with the l1 penalty (Lasso), implemented in R *glmnet* package was performed. The optimal parameter λ was identified by choosing the minimum over a grid and subpathway signatures with non-zero coefficients were selected.

### A drug-subpathway network was constructed based on HGCC resource

Based on the HGCC resource [28], we obtained the drug IC50 information, as well as gene expression, methylation and CNV data for each GBM cell line. Firstly, based on the median IC50 value as cutoff, we defined two cell line groups, high IC50 groups and low IC50 groups. And then, based on these two groups, we respectively identified drug related genes based on gene expression level, methylation condition, and CNV data. For gene expression profiles, the T-test was used. And for methylation and CNV data, the wilcoxon rank sum test was used. And the cutoff for differentially expression (DE) analysis was set as adjusted P-value < 0.05. Finally, we evaluated the associations between DE genes and 28 microglia subpathways by using the hypergeometric test method. And the result with P-value < 0.05 was considered as the significant associations. An integrated drug-subpathway network was constructed by considering three level of omics data.

## Supplementary Information

**Additional file 1: Figure S1.** Valcanic map of GSE86573 as an example. The differential expression result in MicT/MacT group (A) and MicT/MicN group (B). Red nodes indicated up-regulated genes, and blue nodes indicated down-regulated genes. Glioma related genes were shown in the maps.

**Additional file 2: Figure S2.** The number of up-regulated and down-regulated genes for MicT/MicN group based on different cutoffs. And the overlapping results among any two data sets was shown.

**Additional file 3: Figure S3**. The framework for constructing microglia specific subpathway network. And the overall framework contained three steps: i) random walk algorithm based on global network to rank candidate genes, ii) identification of microglia specific subpathways, iii) Construct subpathway network.

**Additional file 4: Figure S4.** The verification of top 10 up-regulated and 10 down-regulated genes after random walk analysis from MicT/MacT group, using an independent data set, GSE29949.

**Additional file 5: Figure S5.** The subpathway scores for representative subpathways from MicT/MacT and MicT/MicN groups. And the venn plot shows the associations among these subpathway results.

**Additional file 6: Figure S6.** The microglia subpathway network. The shape reflected the MicT/MacT and MicT/MicN groups. And the color reflected the up-regulated and down-regulated subpathways.

**Additional file 7: Figure S7.** The sankey plot shows the associations between two types of subpathways, total pathway, and pathway classes from KEGG database.

**Additional file 8: Figure S8.** The parameters selection in the Lasso method for identifying SubP28 signature.

**Additional file 9: Figure S9.** The SubP28 score, tumor purity and glioma survival. The correlation relationship between SubP28 score and tumor purity for GBM type (A) and LGG type (B) based on four methods as Fig. 2C. The predictive performance of SubP28 signature in high-purity LGG samples (C) and low-purity LGG sample (D) based on CPE method. (E) The univariate and multivariate cox results of SubP28 score, when further considering sex, grade, age, other clinical factor.

**Additional file 10: Figure S10.** The predictive performance of SubP28 signature in LGG samples without IDH1 mutation.

**Additional file 11: Figure S11.** The SubP28 score of each cell type in two single cell RNA sequencing data, GSE84465 (A) and GSE89567 (B).

**Additional file 12: Figure S12. **The association between SubP28 score and candidate drugs. (A) The associations between SubP28 score and IC50 value of candidate drugs, from three databases including GDSC, HGCC and LENP. The candidate drugs used for treating other tumor types were shown.

**Additional file 13: Figure S13.** The associations of path: 04810_15 activity with glioma molecular subtypes and IDH1 mutation conditions. (A) TCGA GBM dataset. (B) TCGA LGG dataset. (C) GSE72951.

**Additional file 14: Figure S14.** The associations between SubP28 signature and previous glioma functional sets.

**Additional file 15: Table S1.** All the datasets used in the manuscript.

**Additional file 16: Table S2.** The detailed coefficient for each subpathway in SubP28.

## Data Availability

The data that support the findings of this study are available from the corresponding author upon reasonable request.
