# Supplementary figures and images for: Comprehensive analysis of microglia gene and subpathway signatures for glioma prognosis and drug screening: linking microglia to glioma
Source: J Transl Med. 2022 Jun 21;20:277. doi: 10.1186/s12967-022-03475-8 (PMC9210642; doi:10.1186/s12967-022-03475-8)

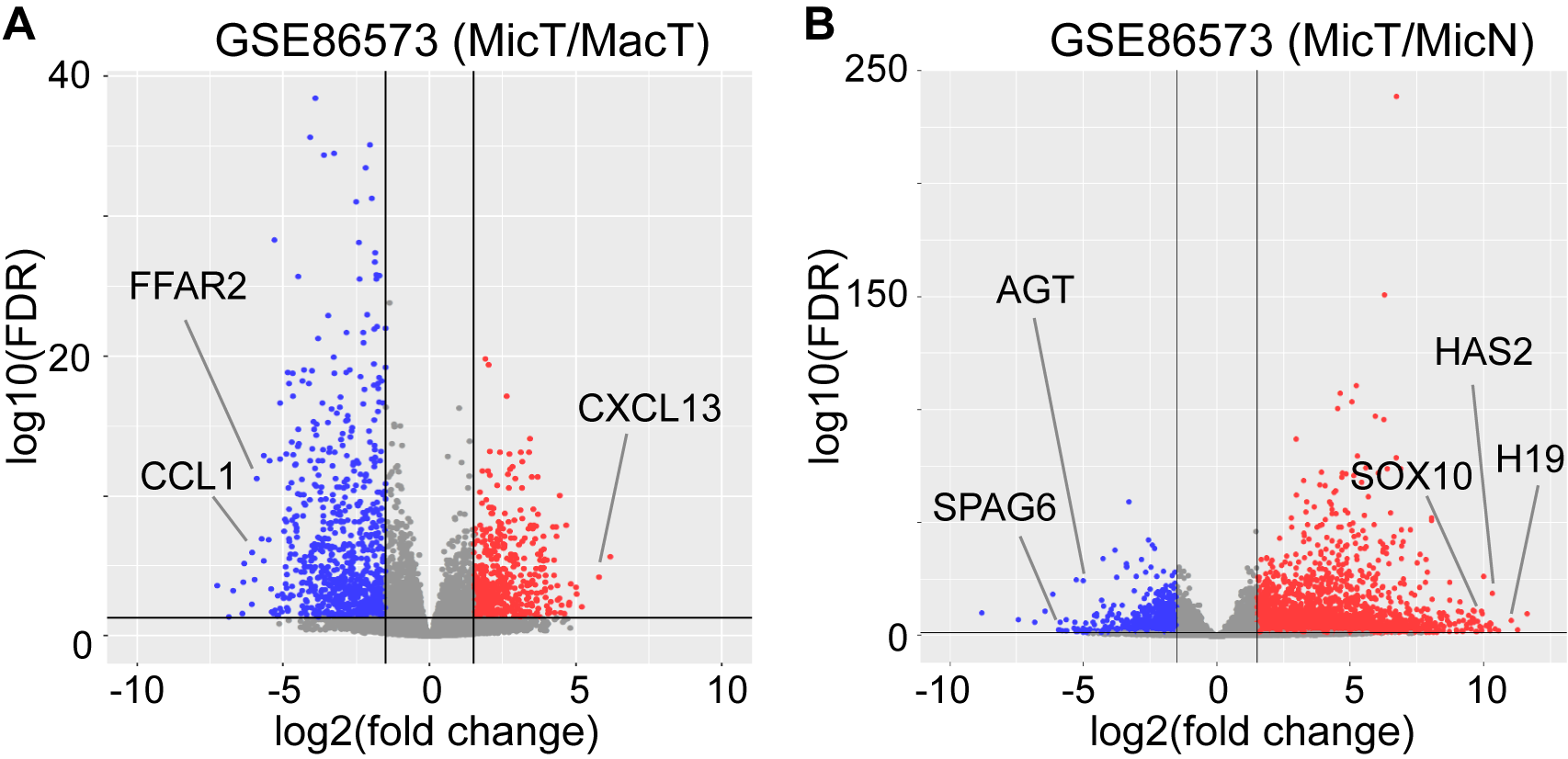

Supplement: Supplementary file 1 — Additional file 1: Figure S1. Valcanic map of GSE86573 as an example. The differential expression result in MicT/MacT group (A) and MicT/MicN group (B). Red nodes indicated up-regulated genes, and blue nodes indicated down-regulated genes. Glioma related genes were shown in the maps. [file 12967_2022_3475_MOESM1_ESM.tif]

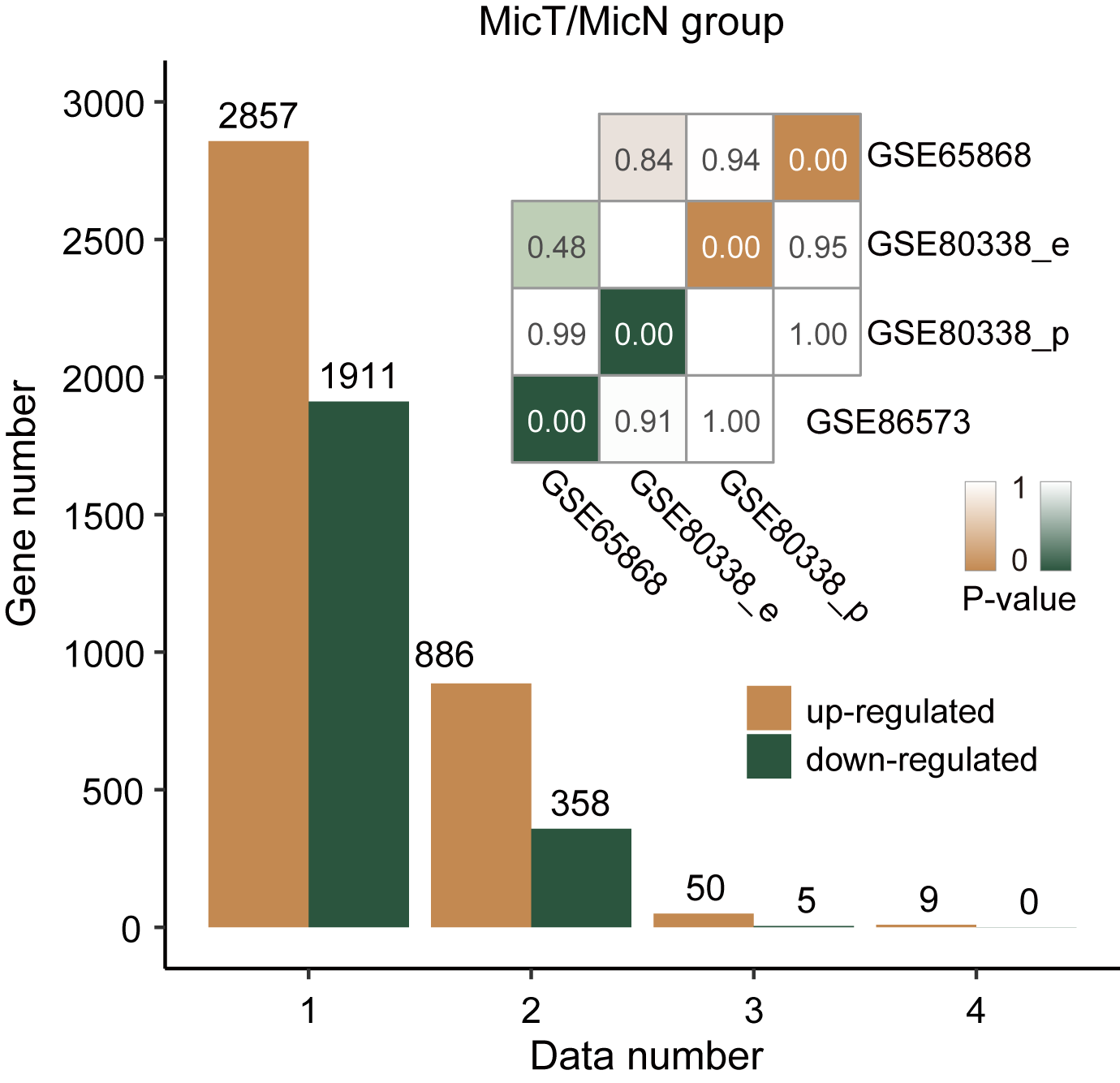

Supplement: Supplementary file 2 — Additional file 2: Figure S2. The number of up-regulated and down-regulated genes for MicT/MicN group based on different cutoffs. And the overlapping results among any two data sets was shown. [file 12967_2022_3475_MOESM2_ESM.tif]

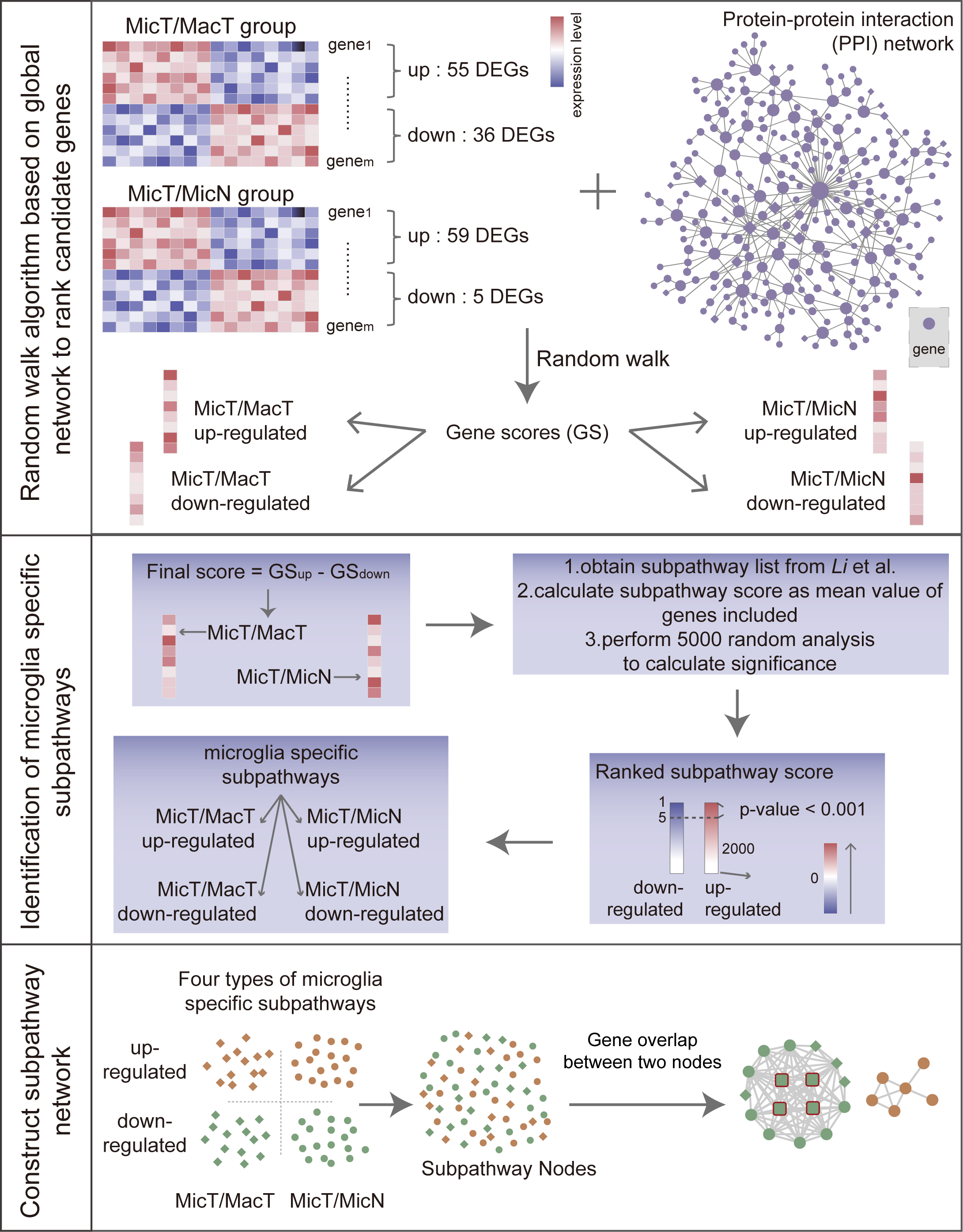

Supplement: Supplementary file 3 — Additional file 3: Figure S3. The framework for constructing microglia specific subpathway network. And the overall framework contained three steps: i) random walk algorithm based on global network to rank candidate genes, ii) identification of microglia specific subpathways, iii) Construct subpathway network. [file 12967_2022_3475_MOESM3_ESM.tif]

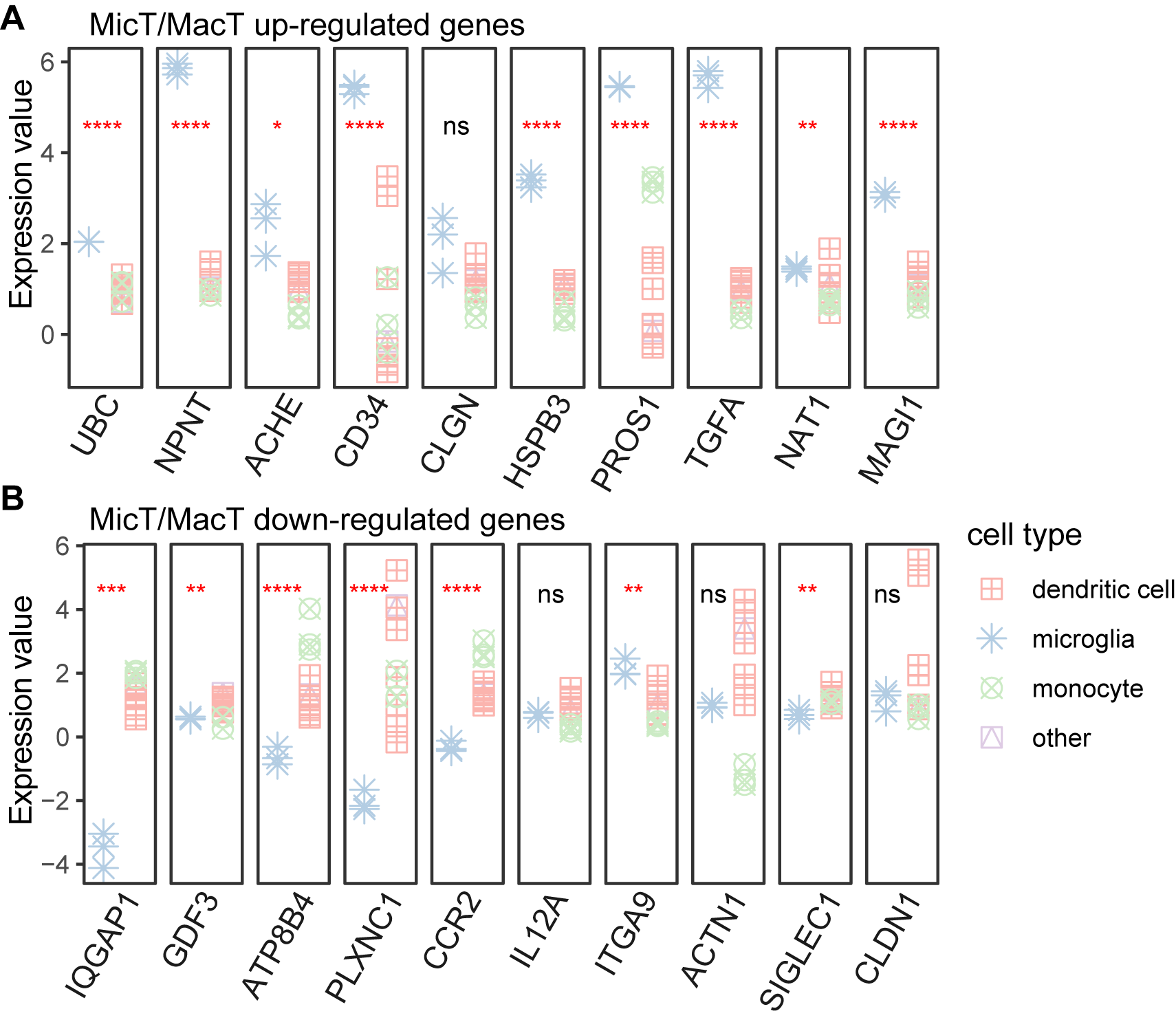

Supplement: Supplementary file 4 — Additional file 4: Figure S4. The verification of top 10 up-regulated and 10 down-regulated genes after random walk analysis from MicT/MacT group, using an independent data set, GSE29949. [file 12967_2022_3475_MOESM4_ESM.tif]

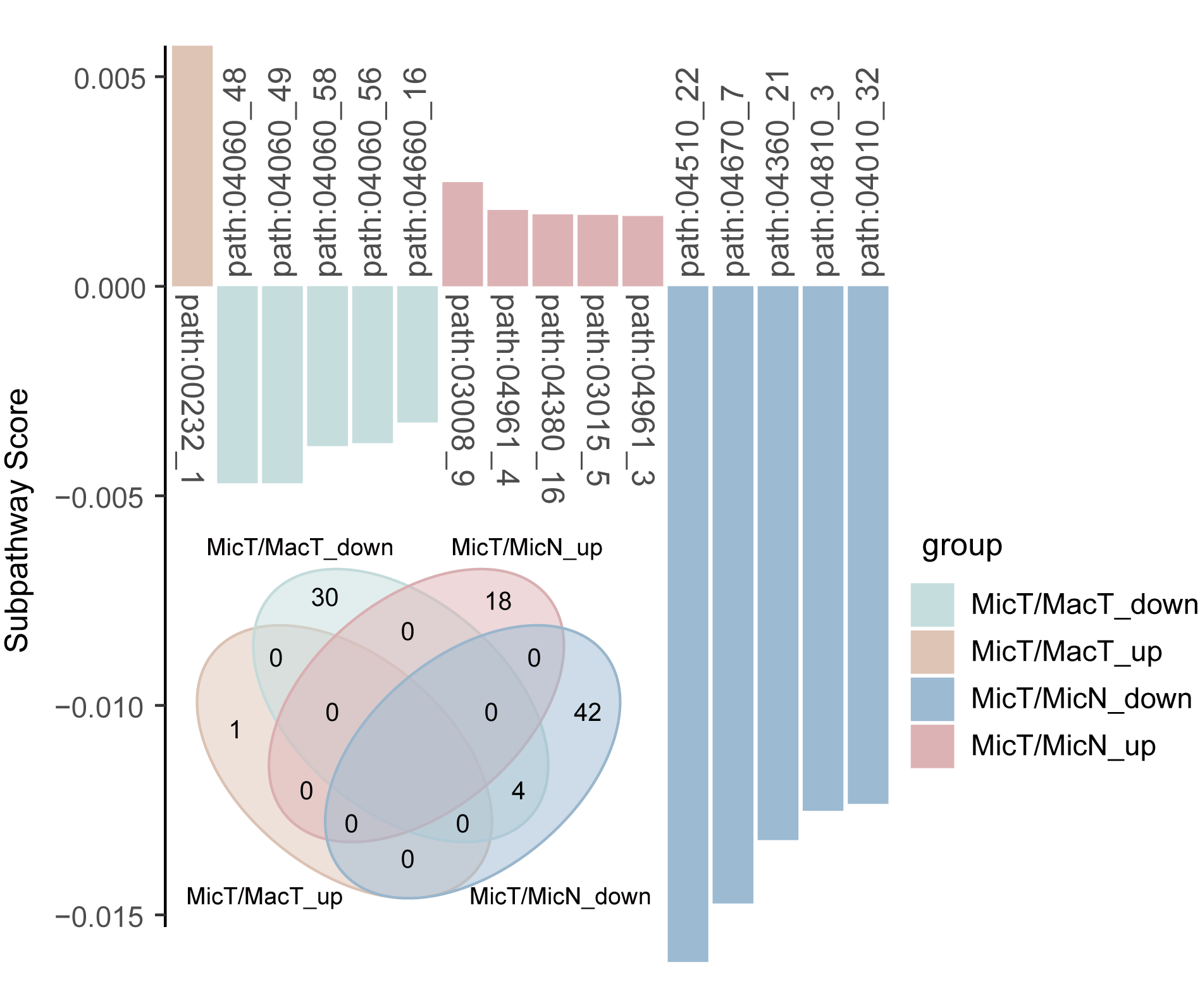

Supplement: Supplementary file 5 — Additional file 5: Figure S5. The subpathway scores for representative subpathways from MicT/MacT and MicT/MicN groups. And the venn plot shows the associations among these subpathway results. [file 12967_2022_3475_MOESM5_ESM.tif]

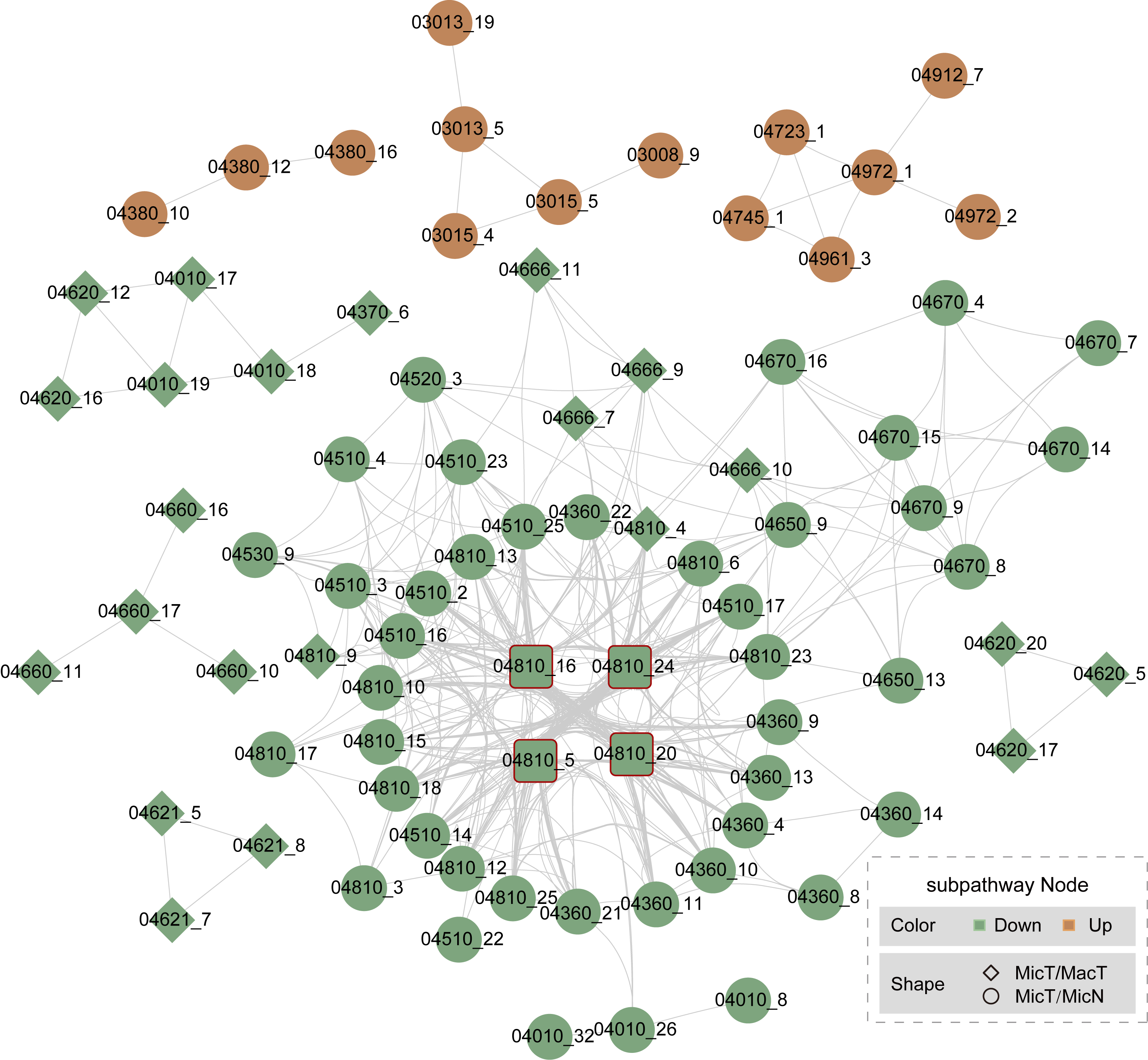

Supplement: Supplementary file 6 — Additional file 6: Figure S6. The microglia subpathway network. The shape reflected the MicT/MacT and MicT/MicN groups. And the color reflected the up-regulated and down-regulated subpathways. [file 12967_2022_3475_MOESM6_ESM.tif]

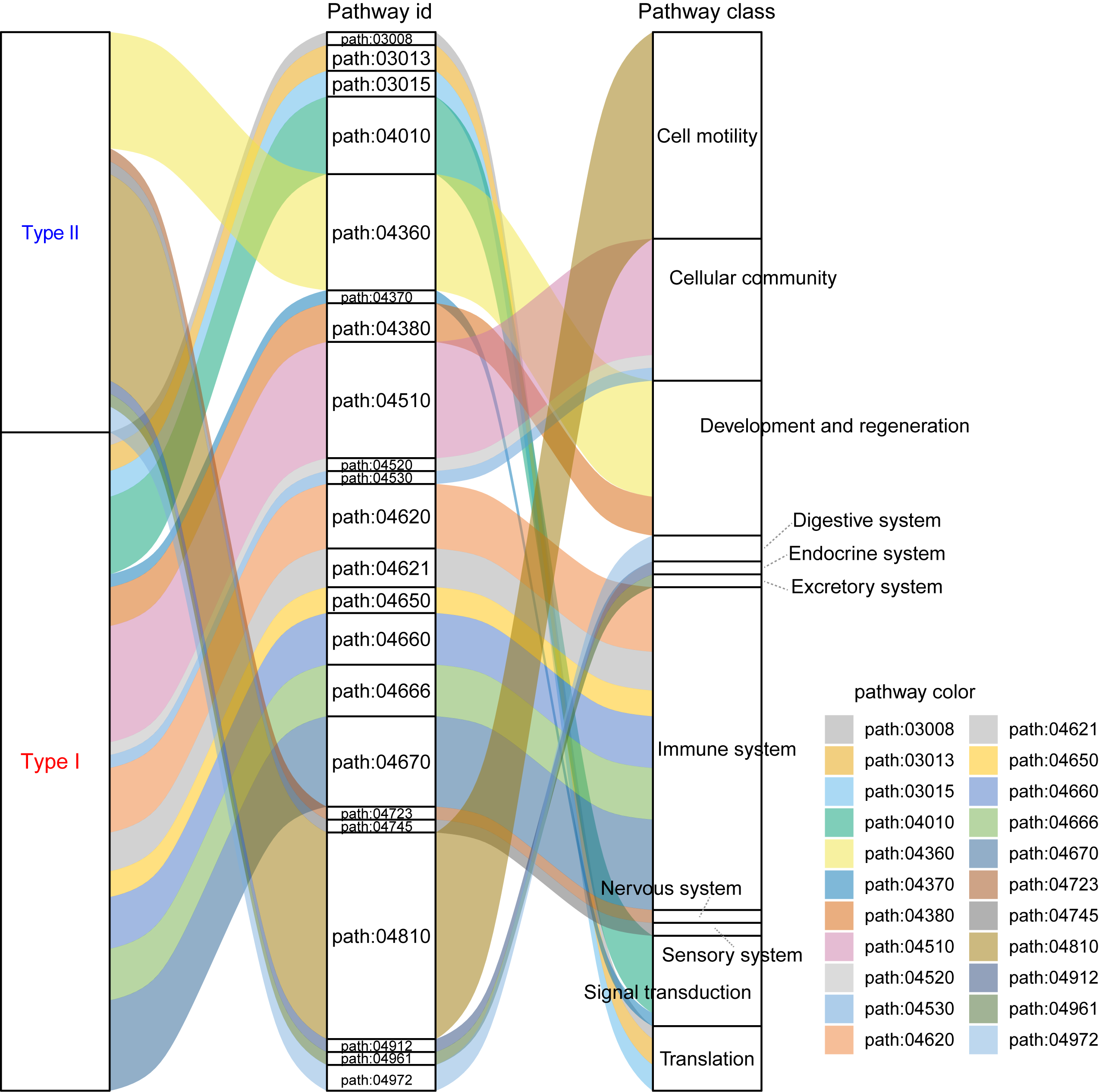

Supplement: Supplementary file 7 — Additional file 7: Figure S7. The sankey plot shows the associations between two types of subpathways, total pathway, and pathway classes from KEGG database. [file 12967_2022_3475_MOESM7_ESM.tif]

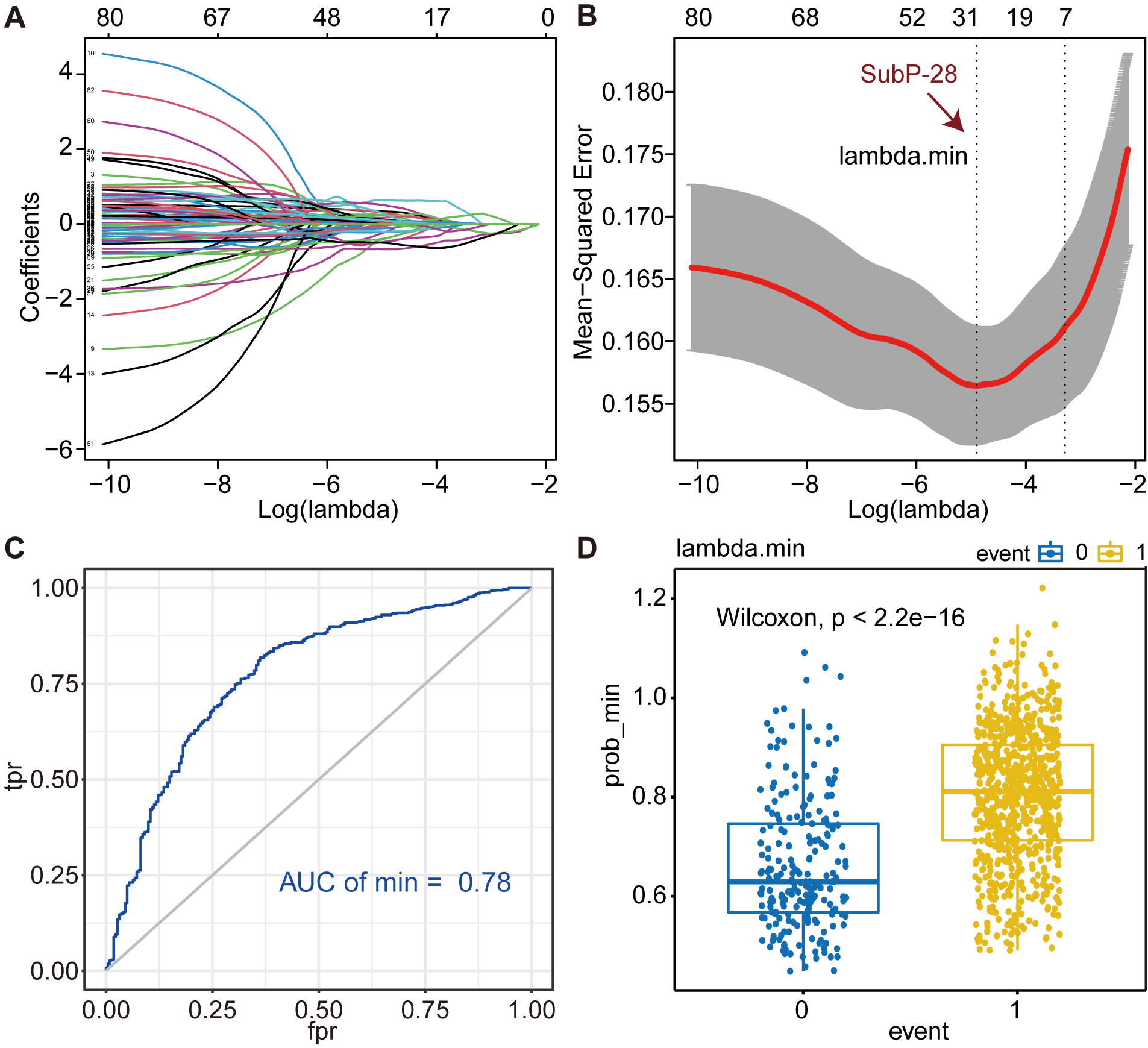

Supplement: Supplementary file 8 — Additional file 8: Figure S8. The parameters selection in the Lasso method for identifying SubP28 signature. [file 12967_2022_3475_MOESM8_ESM.tif]

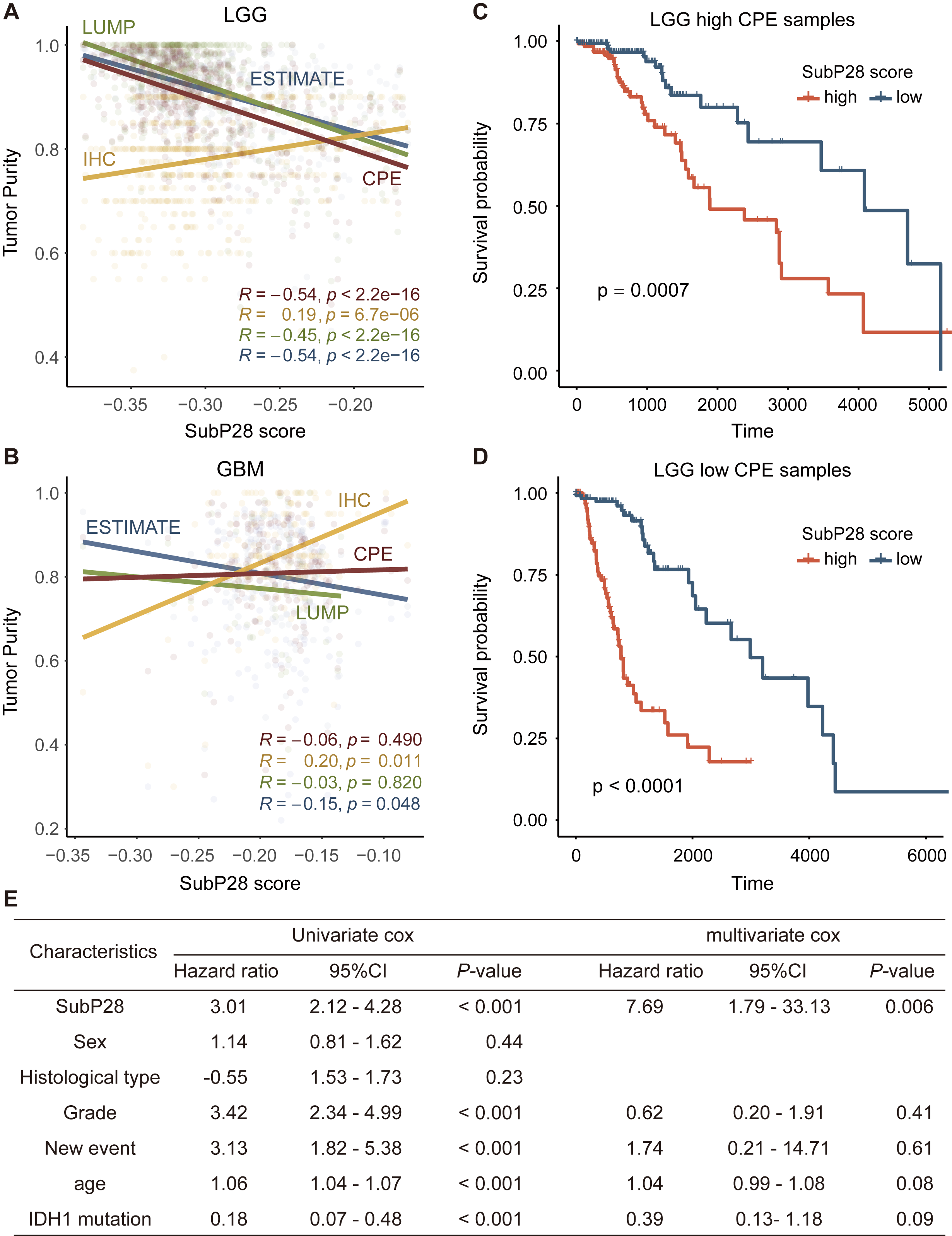

Supplement: Supplementary file 9 — Additional file 9: Figure S9. The SubP28 score, tumor purity and glioma survival. The correlation relationship between SubP28 score and tumor purity for GBM type (A) and LGG type (B) based on four methods as Fig. 2C. The predictive performance of SubP28 signature in high-purity LGG samples (C) and low-purity LGG sample (D) based on CPE method. (E) The univariate and multivariate cox results of SubP28 score, when further considering sex, grade, age, other clinical factor. [file 12967_2022_3475_MOESM9_ESM.tif]

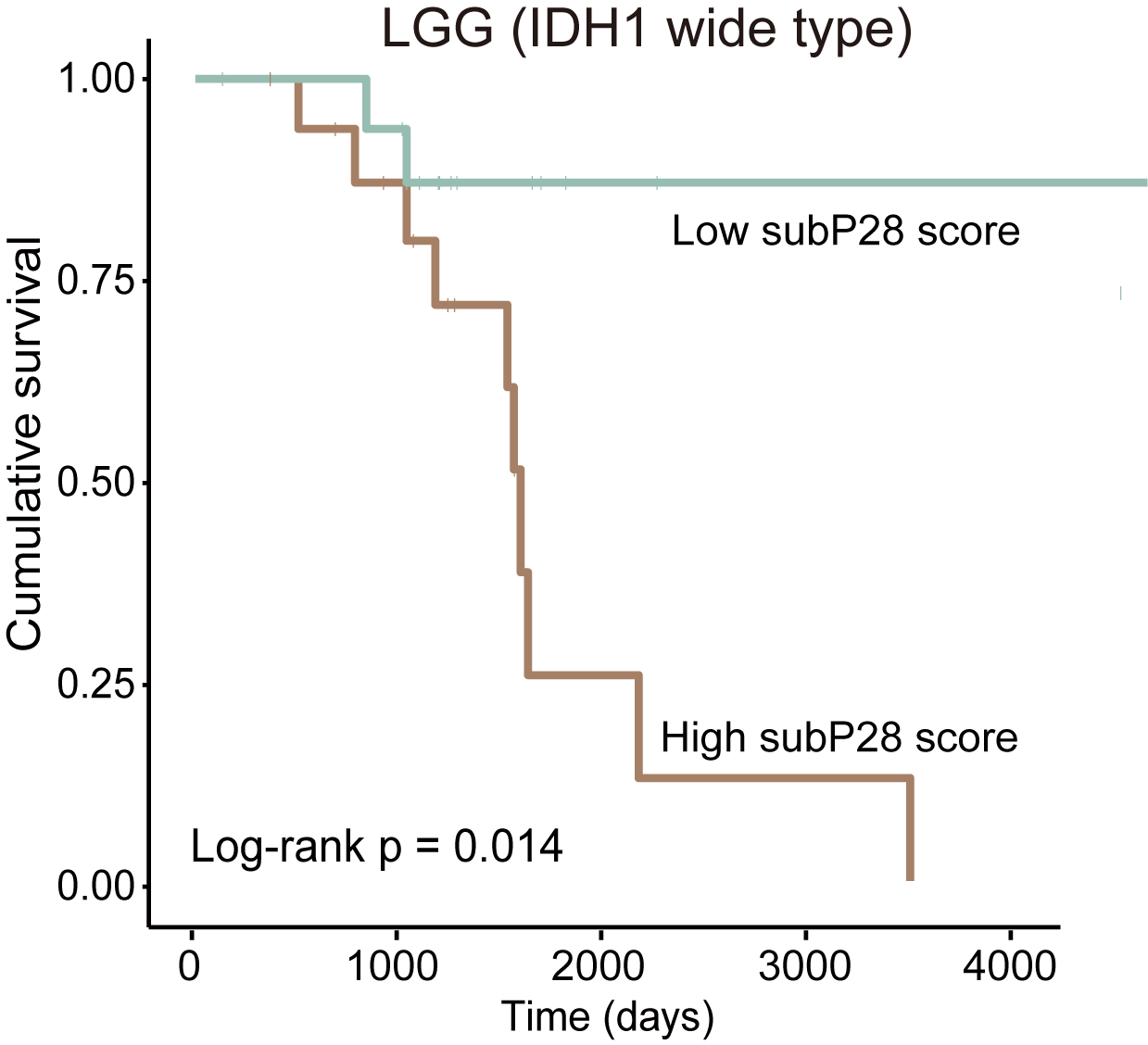

Supplement: Supplementary file 10 — Additional file 10: Figure S10. The predictive performance of SubP28 signature in LGG samples without IDH1 mutation. [file 12967_2022_3475_MOESM10_ESM.tif]

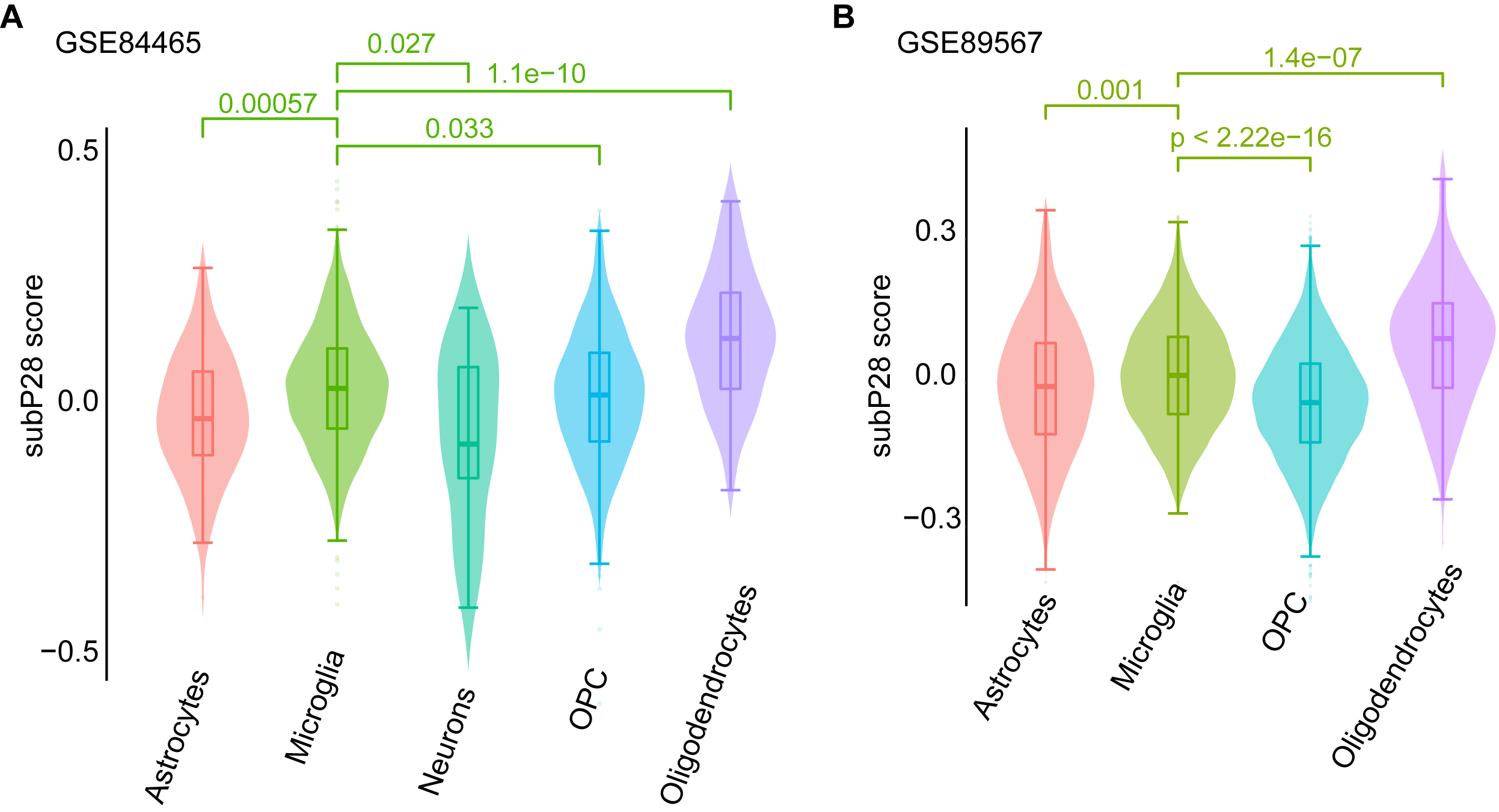

Supplement: Supplementary file 11 — Additional file 11: Figure S11. The SubP28 score of each cell type in two single cell RNA sequencing data, GSE84465 (A) and GSE89567 (B). [file 12967_2022_3475_MOESM11_ESM.tif]

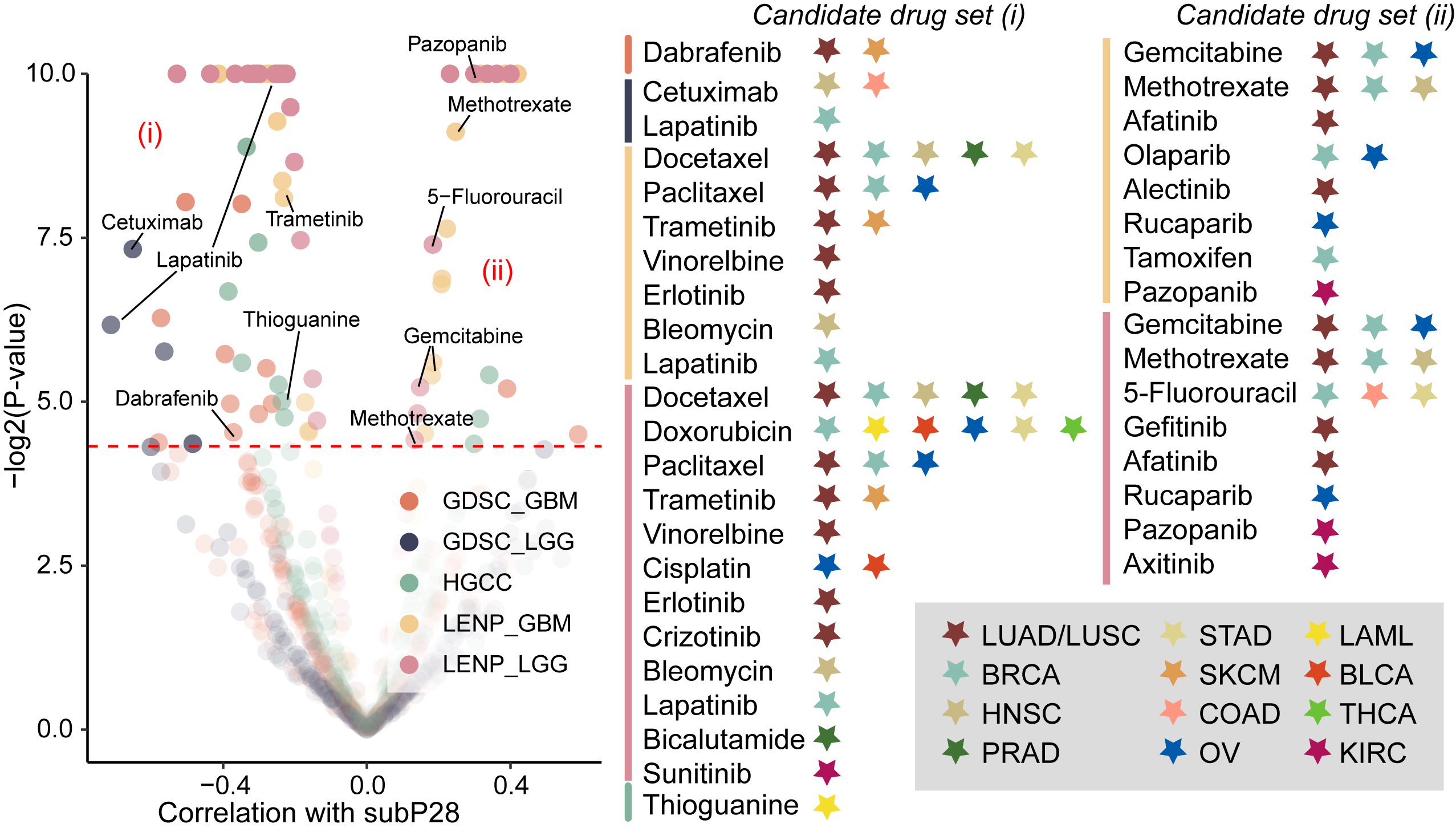

Supplement: Supplementary file 12 — Additional file 12: Figure S12. The association between SubP28 score and candidate drugs. (A) The associations between SubP28 score and IC50 value of candidate drugs, from three databases including GDSC, HGCC and LENP. The candidate drugs used for treating other tumor types were shown. [file 12967_2022_3475_MOESM12_ESM.tif]

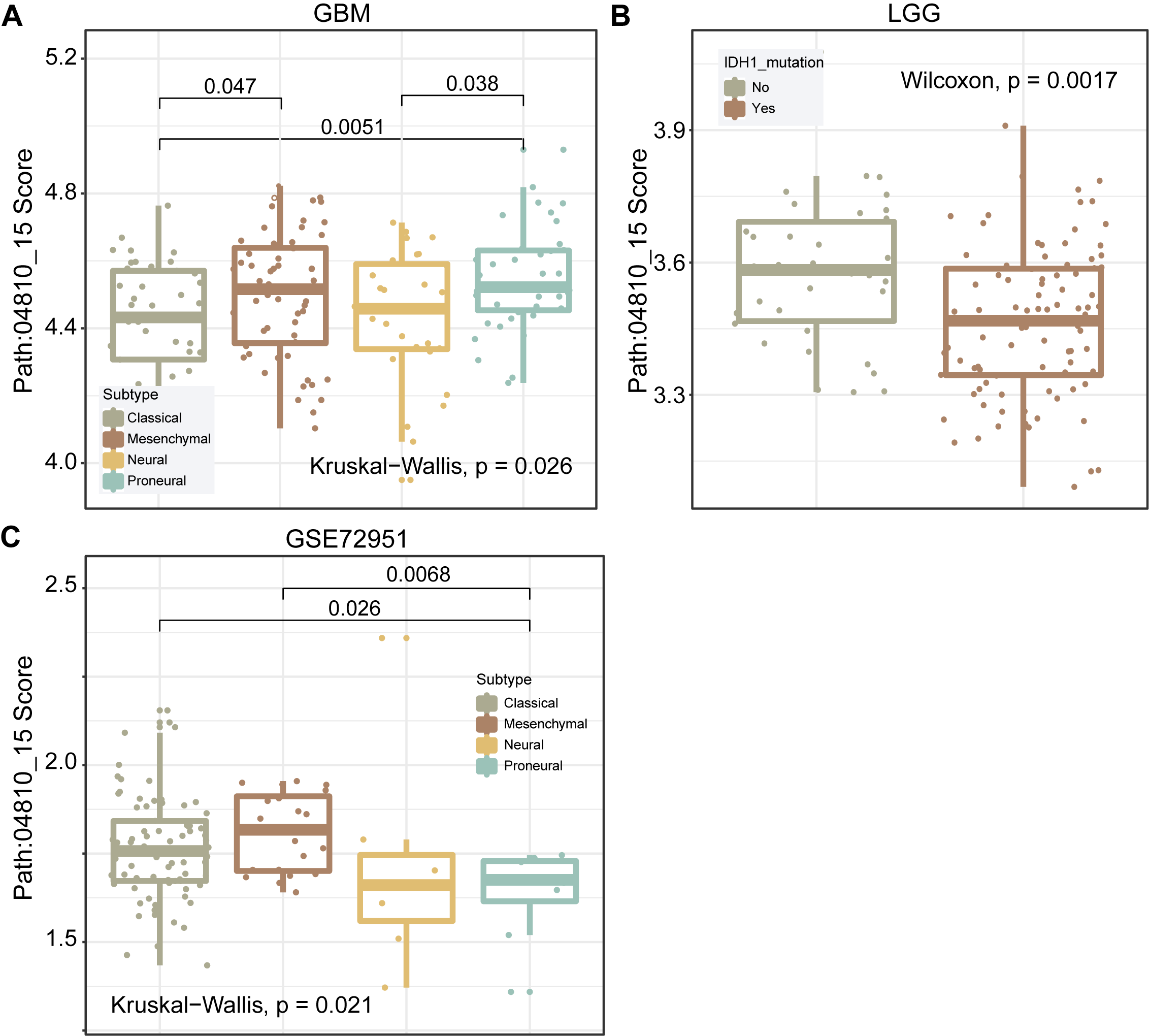

Supplement: Supplementary file 13 — Additional file 13: Figure S13. The associations of path: 04810_15 activity with glioma molecular subtypes and IDH1 mutation conditions. (A) TCGA GBM dataset. (B) TCGA LGG dataset. (C) GSE72951. [file 12967_2022_3475_MOESM13_ESM.tif]

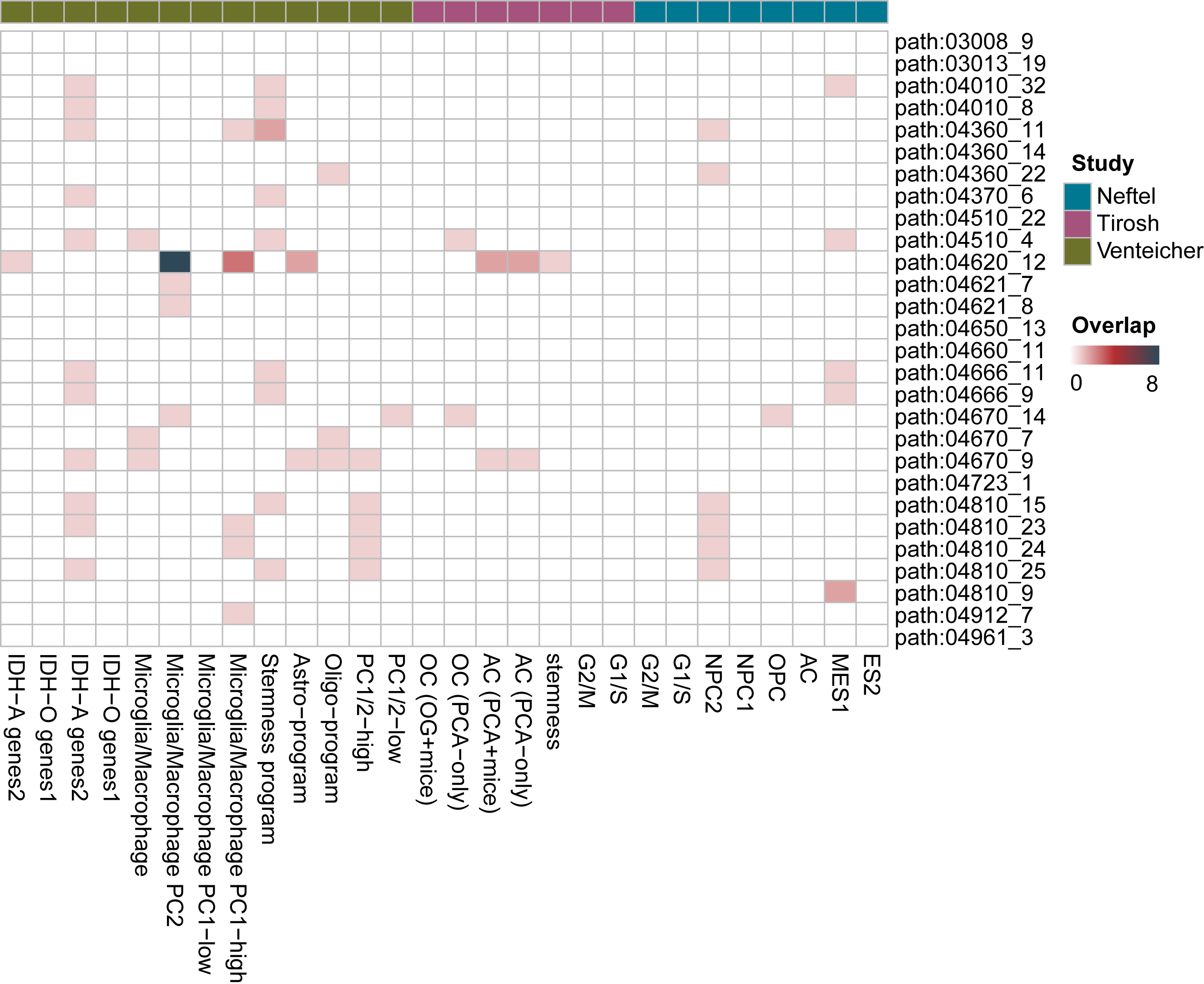

Supplement: Supplementary file 14 — Additional file 14: Figure S14. The associations between SubP28 signature and previous glioma functional sets. [file 12967_2022_3475_MOESM14_ESM.tif]
