# Supplementary material for: Comprehensive analysis of microglia gene and subpathway signatures for glioma prognosis and drug screening: linking microglia to glioma
Source: J Transl Med. 2022 Jun 21;20:277. doi: 10.1186/s12967-022-03475-8 (PMC9210642; doi:10.1186/s12967-022-03475-8)
Supplement: Supplementary file 15 — Additional file 15: Table S1. All the datasets used in the manuscript. [file 12967_2022_3475_MOESM15_ESM.doc]

Table S1. All the datasets used in the manuscript.

| GSE id | Platform | Samples | Usage in this study |
| --- | --- | --- | --- |
| GSE65868 | Illumina HiSeq 2000 | 12 | Microglia characterization analysis |
| GSE86573 | Illumina HiSeq 2000 | 20 | Microglia characterization analysis |
| GSE80338 | Illumina HiSeq 1500 | 16 | Microglia characterization analysis |
| GSE116520 | Illumina HumanHT-12 V4.0 | 42 | Glioma formation |
| GSE15824 | Affymetrix HG U133 Plus 2.0 | 45 | Glioma formation |
| GSE16011 | Affymetrix GeneChip HG U133 Plus 2.0 | 284 | Glioma formation |
| GSE22866 | Agilent-014850 Whole HG Microarray | 46 | Glioma formation |
| GSE35493 | Affymetrix HG U133 Plus 2.0 | 71 | Glioma formation |
| GSE42656 | Illumina HumanHT-12 V3.0 | 73 | Glioma formation |
| GSE4290 | Affymetrix HG U133 Plus 2.0 | 180 | Glioma formation |
| GSE44971 | Affymetrix HG U133 Plus 2.0 | 58 | Glioma formation |
| GSE50021 | Illumina HumanHT-12 WG-DASL V4.0 | 45 | Glioma formation |
| GSE50161 | Affymetrix HG U133 Plus 2.0 | 130 | Glioma formation |
| GSE61335 | Affymetrix HG U133A/B Array | 124 | Glioma formation |
| GSE7696 | Affymetrix HG U133 Plus 2.0 | 84 | Glioma formation |
| GSE101113 | Affymetrix HG U133 Plus 2.0 | 56 | Glioma recurrence |
| GSE62153 | Illumina HumanHT-12 V4.0 | 43 | Glioma recurrence |
| GSE98995 | Affymetrix HG U133 Plus 2.0 | 71 | Glioma recurrence |
| GSE42670 | Affymetrix Human Gene 1.0 ST | 58 | Glioma survival |
| GSE72951 | Illumina HumanHT-12 WG-DASL V4.0 | 112 | Glioma survival |
| GSE74187 | Agilent-014850 Whole HG Microarray | 60 | Glioma survival |
| GSE83300 | Agilent-014850 Whole HG Microarray | 50 | Glioma survival |

A.The Gene Expression Omnibus (GEO) datasets

B.The other glioma gene expression datasets

| Database id | Web Link | Usage in this study |
| --- | --- | --- |
| TCGA-GBM | https://portal.gdc.cancer.gov/ | Glioma formation/Glioma recurrence/Glioma prognosis |
| CGGA | http://www.cgga.org.cn/ | Glioma recurrence/Glioma prognosis |
| TCGA-LGG | https://portal.gdc.cancer.gov/ | Glioma recurrence/Glioma prognosis |
| PCAWG |  | Glioma prognosis |

C.The brain cell line datase

| Database id/Method name | Data Link |
| --- | --- |
| GDSC | https://www.cancerrxgene.org/ |
| LENP supplementary file | https://www.ncbi.nlm.nih.gov/pmc/articles/PMC6085336/ |
| HGCC | portal.hgcc.se |
