# Supplementary material for: Comprehensive analysis of microglia gene and subpathway signatures for glioma prognosis and drug screening: linking microglia to glioma
Source: J Transl Med. 2022 Jun 21;20:277. doi: 10.1186/s12967-022-03475-8 (PMC9210642; doi:10.1186/s12967-022-03475-8)
Supplement: Supplementary file 16 — Additional file 16: Table S2. The detailed coefficient for each subpathway in SubP28. [file 12967_2022_3475_MOESM16_ESM.doc]

Table S2. The detailed coefficient for each subpathway in SubP28.

| **Subpathway id** | **Coefficient** | **Pathway Name** |
| --- | --- | --- |
| path:03008_9 | 0.0182 | Ribosome biogenesis in eukaryotes |
| path:03013_19 | 0.3341 | RNA transport |
| path:04010_32 | 0.0881 | MAPK signaling pathway |
| path:04010_8 | -0.3703 | MAPK signaling pathway |
| path:04360_11 | 0.4893 | Axon guidance |
| path:04360_14 | -0.0379 | Axon guidance |
| path:04360_22 | -0.6750 | Axon guidance |
| path:04370_6 | 0.0844 | VEGF signaling pathway |
| path:04510_22 | -0.5111 | Focal adhesion |
| path:04510_4 | 0.0161 | Focal adhesion |
| path:04620_12 | -0.1082 | Toll-like receptor signaling pathway |
| path:04621_7 | 0.2410 | NOD-like receptor signaling pathway |
| path:04621_8 | -0.1916 | NOD-like receptor signaling pathway |
| path:04650_13 | -0.4736 | Natural killer cell mediated cytotoxicity |
| path:04660_11 | 0.0368 | T cell receptor signaling pathway |
| path:04666_11 | 0.2289 | Fc gamma R-mediated phagocytosis |
| path:04666_9 | 0.2210 | Fc gamma R-mediated phagocytosis |
| path:04670_14 | 0.0130 | Leukocyte transendothelial migration |
| path:04670_7 | 0.0453 | Leukocyte transendothelial migration |
| path:04670_9 | 0.1119 | Leukocyte transendothelial migration |
| path:04723_1 | -0.0075 | Retrograde endocannabinoid signaling |
| path:04810_15 | 0.6327 | Regulation of actin cytoskeleton |
| path:04810_23 | -0.4626 | Regulation of actin cytoskeleton |
| path:04810_24 | -0.3771 | Regulation of actin cytoskeleton |
| path:04810_25 | 0.2894 | Regulation of actin cytoskeleton |
| path:04810_9 | 0.1745 | Regulation of actin cytoskeleton |
| path:04912_7 | -0.2142 | GnRH signaling pathway |
| path:04961_3 | -0.2011 | Endocrine and other factor-regulated calcium reabsorption |
